# Transcriptome-wide analysis reveals potential roles of CFD and ANGPTL4 in fibroblasts regulating B cell lineage for extracellular matrix-driven clustering and novel avenues for immunotherapy in breast cancer

**DOI:** 10.1186/s10020-025-01237-y

**Published:** 2025-05-08

**Authors:** Hongwei Wang, Yu-nan Zhu, Sifan Zhang, Kexin Liu, Rong Huang, Zhigao Li, Lan Mei, Yingpu Li

**Affiliations:** 1 Department of Oncological Surgery, Harbin Medical University Cancer Hospital, Harbin, 150000 Heilongjiang Province China; 2 Department of Neurobiology, Harbin Medical University, Harbin, 150081 Heilongjiang Province China; 3 NHC Key Laboratory of Cell Transplantation, The First Affiliated Hospital of Harbin Medical University, Harbin, 150001 Heilongjiang Province China; 4 Genomics Research Center (Key Laboratory of Gut Microbiota and Pharmacogenomics of Heilongjiang Province), College of Pharmacy, Harbin Medical University, Harbin, 150081 Heilongjiang Province China

**Keywords:** Extracellular matrix, Breast cancer, CFD, ANGPTL4, Immunotherapy

## Abstract

**Background:**

The remodeling of the extracellular matrix (ECM) plays a pivotal role in tumor progression and drug resistance. However, the compositional patterns of ECM in breast cancer and their underlying biological functions remain elusive.

**Methods:**

Transcriptome and genome data of breast cancer patients from TCGA database was downloaded. Patients were classified into different clusters by using non-negative matrix factorization (NMF) based on signatures of ECM components and regulators. Weighted Gene Co-expression Network Analysis (WGCNA) was used to identify core genes related to ECM clusters. Additional 10 independent public cohorts including Metabric, SCAN_B, GSE12276, GSE16446, GSE19615, GSE20685, GSE21653, GSE58644, GSE58812, and GSE88770 were collected to construct Training or Testing cohort, following machine learning calculating ECM correlated index (ECI) for survival analysis. Pathway enrichment and correlation analysis were used to explore the relationship among ECM clusters, ECI and TME. Single-cell transcriptome data from GSE161529 was processed for uncovering the differences among ECM clusters.

**Results:**

Using NMF, we identified three ECM clusters in the TCGA database: C1 (Neuron), C2 (ECM), and C3 (Immune). Subsequently, WGCNA was employed to pinpoint cluster-specific genes and develop a prognostic model. This model demonstrated robust predictive power for breast cancer patient survival in both the Training cohort (*n* = 5,392, AUC = 0.861) and the Testing cohort (*n* = 1,344, AUC = 0.711). Upon analyzing the tumor microenvironment (TME), we discovered that fibroblasts and B cell lineage were the core cell types associated with the ECM cluster phenotypes. Single-cell RNA sequencing data further revealed that angiopoietin like 4 (ANGPTL4)^+^ fibroblasts were specifically linked to the C2 phenotype, while complement factor D (CFD)^+^ fibroblasts characterized the other ECM clusters. CellChat analysis indicated that ANGPTL4^+^ and CFD^+^ fibroblasts regulate B cell lineage via distinct signaling pathways. Additionally, analysis using the Kaplan–Meier Plotter website showed that CFD was favorable for immunotherapy response, whereas ANGPTL4 negatively impacted the outcomes of cancer patients receiving immunotherapy.

**Conclusion:**

We identified distinct ECM clusters in breast cancer patients, irrespective of molecular subtypes. Additionally, we constructed an effective prognostic model based on these ECM clusters and recognized ANGPTL4^+^ and CFD^+^ fibroblasts as potential biomarkers for immunotherapy in breast cancer.

**Supplementary Information:**

The online version contains supplementary material available at 10.1186/s10020-025-01237-y.

## Introduction

The remodeling of the extracellular matrix (ECM) in cancer plays a crucial role in driving disease progression and often leads to drug resistance and unfavorable outcomes for patients. Mechanistically, the intricate composition of the ECM gives rise to diverse ligand-receptor interactions and downstream signaling pathways (Cox 2021). Furthermore, ECM remodeling alters tissue stiffness, which in turn activates or inhibits specific signaling pathways through mechanotransduction. These phenotypic changes are intertwined with hypoxia (Gilkes et al. 2014), metabolism (Kim et al. 2022), and other factors, fostering cancer cell proliferation, migration, and invasion while impeding the infiltration of immune cells and therapeutic agents. Therefore, targeting the ECM to enhance anti-tumor therapies, such as immunotherapy, has been extensively explored in various malignancies, and present the potential for clinical applications (Karamanos et al. 2021).

Breast cancer, being a superficial organ tumor, often exhibits distinct imaging features that reflect ECM alterations. For instance, high mammographic density stroma is associated with ECM accumulation and is an early event in tumorigenesis, potentially controlled by metabolic reprogramming (DeFilippis et al. 2012; Conner et al. 2024; Bismeijer et al. 2020). Furthermore, ECM aging influences breast cancer phenotypes, including invasion and metastasis (Bahcecioglu et al. 2021; Yang et al. 2024). Our previous works have delved into the current understanding of ECM remodeling in cancer (Yuan et al. 2023; Ye et al. 2023), constructed an effective prognostic model for predicting hormone therapy and neoadjuvant chemotherapy benefits based on ECM signatures in breast cancer (Liu et al. 2022). Importantly, we revealed the predictive value of serum ECM biomarkers in triple-negative breast cancer (TNBC) immunotherapy response (Li et al. 2024a). These results highlights the pivotal role of ECM in regulating response to therapies, especially immunotherapy.

However, it remains uncertain whether different molecular subtypes of breast cancer exhibit distinct ECM profiles and their specific impact on the immune microenvironment, which may contribute to the clinical application of immunotherapy in breast cancer. To address this gap, we conducted a comprehensive investigation into the varying expression levels of ECM components and their regulators in breast cancer. Using data from multiple databases, we identified distinct ECM clusters within breast cancer and characterized their biological processes. Importantly, we focused on the role of ECM in the immune microenvironment, particularly the interplay between ECM clusters and specific cell types such as fibroblasts and B cells. Through this study, we aimed to gain new insights into the role of ECM in breast cancer and identify potential biomarkers for immunotherapy.

## Method

### Bulk transcriptome data collection and integration

We obtained RNA-seq count data from TCGA-BRCA, normalized RNA-seq data from GSE96058 (SCAN_B) (Brueffer et al. 2018), and microarray data from Metabric. Furthermore, we aggregated microarray data from various sources of Gene Expression Omnibus (GEO), including GSE12276 (Bos et al. 2009), GSE16446 (Juul et al. 2010), GSE19615 (Li et al. 2010), GSE20685 (Kao et al. 2011), GSE21653 (Sabatier et al. 2011), GSE58644 (Tofigh et al. 2014), GSE58812 (Jezequel et al. 2015), and GSE88770 (Metzger-Filho et al. 2013), to establish the GEO cohort. Patients lacking overall survival (OS) records were excluded from our analysis. Using these cohorts, we compiled a meta-data cohort consisting of 6,736 breast cancer patients. For bioinformatic analysis of the transcriptome data, we used R (version 4.3). To mitigate batch effects, we applied the combat function from the R package"sva"(version 3.50.0) (Leek et al. 2012). Additionally, the R package"IOBR"(version 0.99.8) was utilized for transcript per million (TPM) normalization and Principal Component Analysis (PCA) (Zeng et al. 2021).

### Non-negative matrix factorization (NMF) analysis

In this work, tsv. data of gene sets of collagens (NABA_COLLAGENS.v2024.1.Hs), glycoproteins (NABA_ECM_GLYCOPROTEINS.v2024.1.Hs), proteoglycans (NABA_PROTEOGLYCANS.v2024.1.Hs), ECM regulators (NABA_ECM_REGULATORS.v2024.1.Hs), and matrisome (NABA_CORE_MATRISOME.v2024.1.Hs) was obtained from the MsigDB database (https://www.gsea-msigdb.org/gsea/msigdb/index.jsp). To further investigate the ECM components and regulators involved in outcome of breast cancer patients, we employed the NMF algorithm (version 0.28) to perform dimensionality reduction analysis on these ECM-related genes (Gaujoux and Seoighe 2010). We determined the optimal value of k, representing the number of clusters, by considering cophenetic correlation coefficients and silhouette scores.

### Differential expression gene and pathway enrichment analysis

For the RNA-seq data, we analyzed differentially expressed genes (DEGs) using the"DESeq2"package (version 1.42.1) in R (Love et al. 2014). For the microarray data, we utilized the"limma"package (version 3.58.1) to identify DEGs (Ritchie et al. 2015). In both cases, genes were considered DEGs if they had an adjusted p-value of less than 0.01 and an absolute log_2_ fold change (FC) greater than 1. After identifying these DEGs, we performed a comprehensive pathway analysis of plasma proteins using the Molecular Signatures Database ("msigdb", version 7.5.1), which encompassed HALLMARK and other pathways (Liberzon et al. 2015). Additionally, we conducted Reactome pathway analysis with the"ReactomePA"package (version 1.46.0) in R (Yu and He 2016). For further exploration, we performed Gene Set Variation Analysis (GSVA) using the"clusterProfiler"package (version 4.12.3) (Wu et al. 2021).

### Weighted Gene Co-expression Network Analysis (WGCNA) of ECM clusters in breast cancer patients

The “WGCNA” package (version 1.73) was utilized to establish a gene co-expression network based on a selected set of 5,000 top variable genes (Langfelder and Horvath 2008). Subsequently, hierarchical clustering analysis was conducted to identify modules within the network, employing cut-off values of a minimum modularity of 30 and a merge height of 0.25. Using the"WGCNA"package, we then evaluated the strength of interactions, calculated gene significance (GS) and module membership (MM), and assessed the correlation between modules and clinical traits. Genes that exhibited high GS and MM within the modules of interest were deemed key genes and were chosen for further analysis. Lastly, the"Pheatmap"package (version 1.0.12) was employed to visually represent the relationship between the modules and ECM clusters.

### Prognostic model construction and survival analysis

A total of 6,736 breast cancer patients, for whom outcome information was available, were randomly assigned to either a Training cohort (*n* = 5392) or a Testing cohort (*n* = 1344) in an 8:2 ratio. To identify the most precise prognostic model within the Training cohort, we leveraged the R package"Mime1"(version 0.0.0.9000), which encompasses 10 distinct machine-learning algorithms (Liu et al. 2024). Genes with the *P* value < 0.05 for Cox regression survival analysis were selected for further prognostic model construction. Subsequently, we employed the random forest (RSF) algorithm to develop a prognostic model. The risk score generated by this model, used to predict the OS of each patient, was designated as the ECM index (ECI). Risk scores were then computed for all patients in the metadata, and patients were stratified into ECI^High^ and ECI^Low^ groups based on the mean ECI value within each cohort. Kaplan–Meier curves were generated to visually compare survival outcomes between these groups, utilizing the"survival"(version 3.7–0) and"survminer"(version 0.4.9) packages.

Univariate and multivariate Cox regression analyses were performed to investigate the association between the ECM index (ECI) and various clinical variables, including age, T stage, N stage, and tumor grade, using the"forestmodel"package (version 0.6.2). Based on the results of these analyses, we identified independent factors that predict OS. Utilizing the"rms"package (version 6.7–0), we generated a nomogram to predict OS for patients, along with a plot of the calibration curve, based on these identified factors. Decision curve analysis (DCA) was conducted and visualized using the"ggDCA"package (version 1.2). To assess the performance of the nomogram, we evaluated the area under the curve (AUC) of the receiver operating characteristic (ROC) curve and diagnostic accuracy. Additionally, survival analysis for immunotherapy was conducted using the Kaplan–Meier Plotter website (kmplot.com) (Kovacs et al. 2023).

### Protein–protein interaction (PPI) and Tumor microenvironment analysis

The protein–protein interaction (PPI) analysis of ECI genes was performed utilizing the STRING dataset (version 12.0, available at https://string-db.org/). The interacting proteins identified within each cluster were subsequently subjected to Reactome analysis, and the five most enriched pathways were chosen for visualization. Cystoscope (version 3.9.1) was then used to create visual representations of the PPI network, highlighting the importance and pathway enrichment of the ECI genes. To determine the infiltration score of various TME components, we utilized the R package"IOBR"(version 0.99.8), which integrates multiple algorithms including Cibersort, EPIC, Estimate, IPS, MCP_counter, TIMER, and xCell (Zeng et al. 2021).

### Correlation analysis and mantel testing analysis

The correlation between individual TME components and the ECI was evaluated using Spearman’s correlation method. The results were visually represented using the R packages"corrplot"(version 0.92) and"ggcorrplot"(version 0.1.4.1). A Spearman correlation coefficient with an absolute value (|R|) greater than 0.4 and a p-value less than 0.05 were considered as thresholds for indicating a statistically significant correlation. Additionally, the Mantel Testing results were explored using the"linkET"package (version 0.0.7.4).

### Single-Cell RNA sequencing data integration and analysis

For this study, single-cell RNA sequencing (scRNA-seq) data from GSE161529 were obtained from the GEO database. This dataset encompassed scRNA-seq data derived from 31 primary breast tumor samples. The"Seurat"package (version 4.4) was employed to integrate the scRNA-seq data (Satija et al. 2015). Cells with a gene expression count below 200 or exceeding 5000 were excluded from the analysis. Furthermore, cells with a mitochondrial content greater than 25% were also filtered out, leaving a total of 210,293 cells with single-cell transcriptome data for subsequent analysis. To address batch effects within the dataset GSE161529, the"harmony"package (version 1.2.1) was utilized (Korsunsky et al. 2019). Firstly, the data was normalized by using the “LogNormalize” function, followed by the “Scale” to regress out the cell cycle scores ("S.Score"and G2M.Score. Then, the data was integrated across different batches or conditions using RunHarmony, specifying the batch variable by each sample. Computing UMAP based on the Harmony-integrated data using “RunUMAP”, specifying dimensions 1 to 25 for the reduction. Finding neighbors in the Harmony-integrated space using “FindNeighbors”, specifying dimensions 1 to 20. And then cells were clustered at multiple resolutions using FindClusters, setting a sequence of resolutions from 0 to 1 by 0.2 intervals to observe clustering effects at different granularities. Cell annotation was performed based on the CellMarker 2.0 database (Hu et al. 2023). Additionally, the ligand-receptor interactions between different cell clusters were quantified using the R package"CellChat"(version 2.1.2) (Jin et al. 2024). And Scissor algorithm was used to link bulk RNA-seq and scRNA-seq (Sun et al. 2022).

## Results

### NMF-based classification of breast cancer subtypes based on ECM components and regulators reveals distinct survival outcomes

**Fig. 1:**
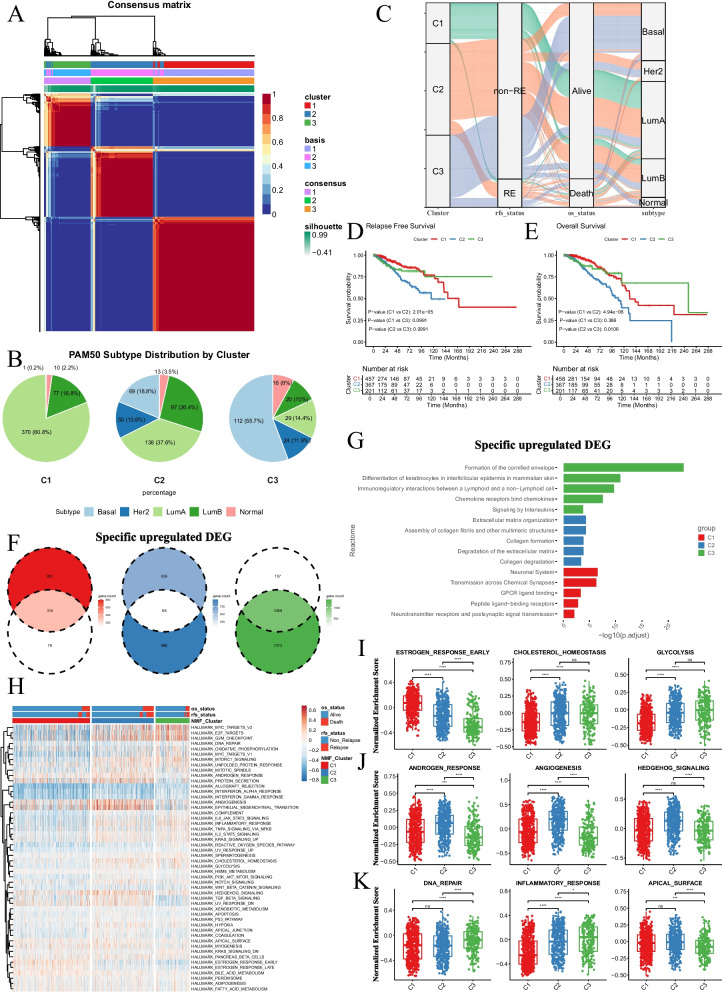
NMF clustering breast cancer patients base on ECM signatures. **A** Heatmap plotting three ECM clusters based on NMF algorism in TCGA-BRCA cohort. **B** Circle plot illustrating the distribution of molecular subtypes of breast cancer in each ECM clusters. **C** Sangi plot illustrating the linkage among ECM clusters, recurrence survival status, OS status, and molecular subtypes of breast cancer. **D** K-M curve plotting RFS status of patients with each ECM clusters. **E** K-M curve plotting OS status of patients with each ECM clusters. **F** Venn diagrams showing the intersection of upregulated DEGs obtained from the comparation among each ECM clusters. **G** Box plot illustrating the top 5 enriched Reactome pathways of specific upregulated DEGs in each ECM cluster. **H** Heatmap plotting the enrichment status of HALLMARK gene sets in each ECM cluster. **I**-**K** Box plot illustrating the enrichment status of HALLMARK gene sets in C1 (**I**), C2 (**J**), C3(**K**). Wilcox test, *: *P* < 0.05, **: *P* < 0.01, ***: *P* < 0.001, ****: *P* < 0.0001

To delve into the diverse composition of the ECM in breast cancer, we curated a panel of 513 signatures encompassing collagens, glycoproteins, proteoglycans, ECM regulators, and other ECM-related components. Utilizing NMF algorithm, we classified 1026 patients from the TCGA-BRCA dataset based on the transcriptional expression of these signatures. Through optimal NMF measurements and analysis, we identified three distinct clusters (Fig. 1, S1, and Table 1). In terms of molecular classification, patients in cluster 1 (C1) predominantly exhibited Luminal subtype characteristics, while those in cluster 3 (C3) were largely estrogen-independent, such as TNBC or Her-2 positive (HER2) (Fig. 1B, and Table S1). Notably, cluster 2 (C2) encompassed a moderate proportion of each molecular subtype (Fig. 1B) and was associated with significantly poorer outcomes compared to the other two clusters (Fig. 1C-E). These findings suggest that specific ECM compositions and regulators may predict poor outcomes in breast cancer patients, irrespective of their molecular subtype.

**Table 1:** Clinical information of each ECM cluster in TCGA-BRCA cohort

	Level	C1	C2	C3	P
n		458	367	201	
Subtype (%)	Basal	1 (0.2)	69 (18.8)	112 (55.7)	< 0.001
	Her2	0 (0.0)	50 (13.6)	24 (11.9)	
	LumA	370 (80.8)	138 (37.6)	29 (14.4)	
	LumB	77 (16.8)	97 (26.4)	20 (10.0)	
	Normal	10 (2.2)	13 (3.5)	16 (8.0)	
T_stage (%)	T1	141 (30.8)	90 (24.5)	48 (23.9)	0.001
	T2	242 (52.8)	240 (65.4)	131 (65.2)	
	T3	75 (16.4)	37 (10.1)	22 (10.9)	
N_stage (%)	LN_Negative	224 (49.6)	161 (44.7)	109 (55.1)	0.061
	LN_Positive	228 (50.4)	199 (55.3)	89 (44.9)	
Age (%)	< = 35	7 (1.5)	15 (4.1)	11 (5.5)	0.003
	> = 60	235 (51.3)	161 (43.9)	78 (38.8)	
	35 ~ 60	216 (47.2)	191 (52.0)	112 (55.7)	

Next, we explored the biological characteristics distinguishing these clusters. DEG analysis revealed 315, 68, and 1,089 specifically upregulated DEGs, as well as 887, 14, and 425 specifically downregulated DEGs in C1, C2, and C3, respectively (Fig. 1F, S1B, and S1 C). Reactome pathway analysis indicated that C1 exhibited activation of nerve-associated signaling, C2 was characterized by enhanced ECM remodeling, and C3 showed increased immune response (Fig. 1G and S1D). Additionally, GSVA analysis based on the HALLMARK gene set further distinguished the biological characteristics of each cluster (Fig. 1H). C1 demonstrated higher enrichment of hormonal pathways, such as Estrogen Response Early and Estrogen Response Late, consistent with its predominantly Luminal subtype composition (Fig. 1I and S1E). In contrast, C2 exhibited enhanced enrichment of the Androgen Response pathway (Fig. 1J). Furthermore, several pathways previously reported as ECM-associated, including Angiogenesis, Hedgehog Signaling, and Epithelial-Mesenchymal Transition, were significantly enriched in C2 (Fig. 1J and S1 F). Lastly, C3 was characterized by heightened immune response (Fig. 1K and S1G). These findings underscore the global linkage between ECM and various biological features, ultimately influencing patient outcomes and therapeutic strategies.

### Analysis of breast cancer patient genomes reveals distinct characteristics of ECM clusters

**Fig. 2:**
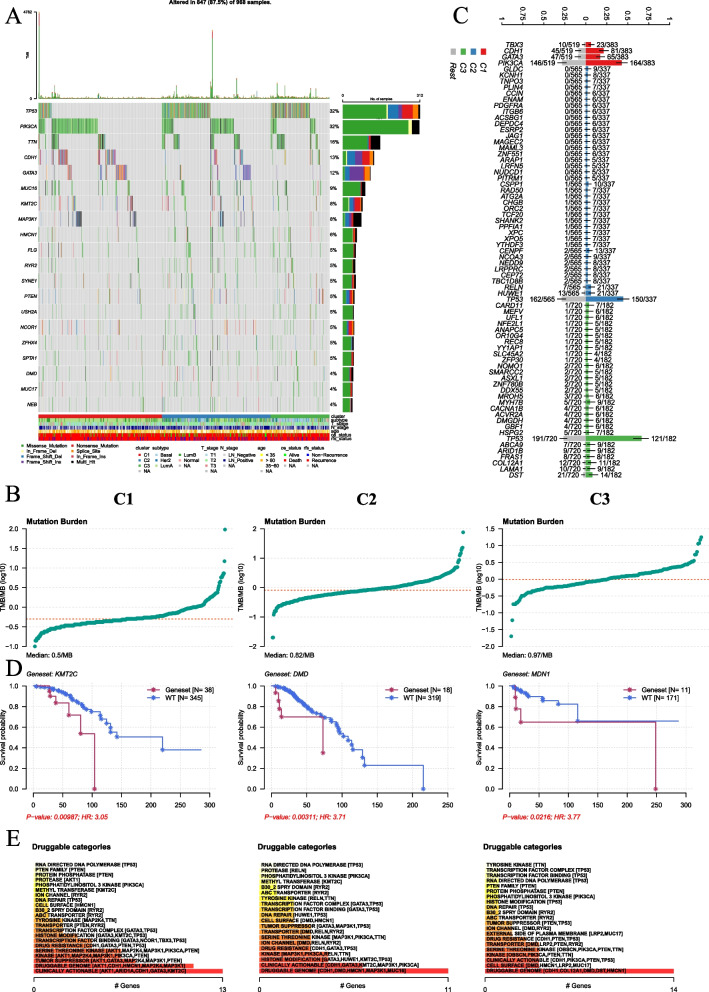
Genomic analysis of breast cancer patients in ECM clusters. **A** Heatmap plotting the most mutant genes in each ECM clusters. **B** Dot plot demonstrating the tumor mutation burden status in each ECM clusters. **C** Box plot illustrating the top differential mutant genes in each ECM clusters in TCGA-BRCA cohort. **D** K-M curve plotting OS status of different ECM cluster patients with *KMT2 C*, *DMD* or *MDN1* mutation. **E** Box plot illustrating the druggable analysis based on mutant genes in each ECM clusters

Subsequently, we examined the varying states of single nucleotide variants (SNVs) within each ECM cluster of breast cancer patients (Fig. 2A and Supplementary Fig. 2 A). Notably, C3 exhibited the highest tumor mutation burden (TMB) compared to the other clusters, while C1 demonstrated the lowest (Fig. 2B). In terms of gene mutations, phosphatidylinositol-4,5-bisphosphate 3-kinase catalytic subunit alpha (PIK3 CA) was predominantly mutated in C1, whereas C2 and C3 harbored more mutations in TP53 (Fig. 2C, S2 A, and S2B). Additionally, a significant mutual exclusivity was observed between TP53 and PIK3 CA mutations in C2 and C3 patients, but not in C1 (Figure S2 C). Survival analysis based on individual gene mutations revealed that mutations in lysine Methyltransferase 2 C (KMT2 C), dystrophin (DMD), and midasin AAA ATPase 1 (MDN1) were associated with poor survival in C1, C2, and C3, respectively (Fig. 2D). Analysis of druggable target genes indicated no significant differences in drug selection among the three clusters. These clusters were defined by their ECM signatures (Fig. 2E). This suggests the need for further exploration at the transcriptome level to identify specific biomarkers.

### WGCNA analysis uncovers core DEGs associated with ECM clusters

**Fig. 3:**
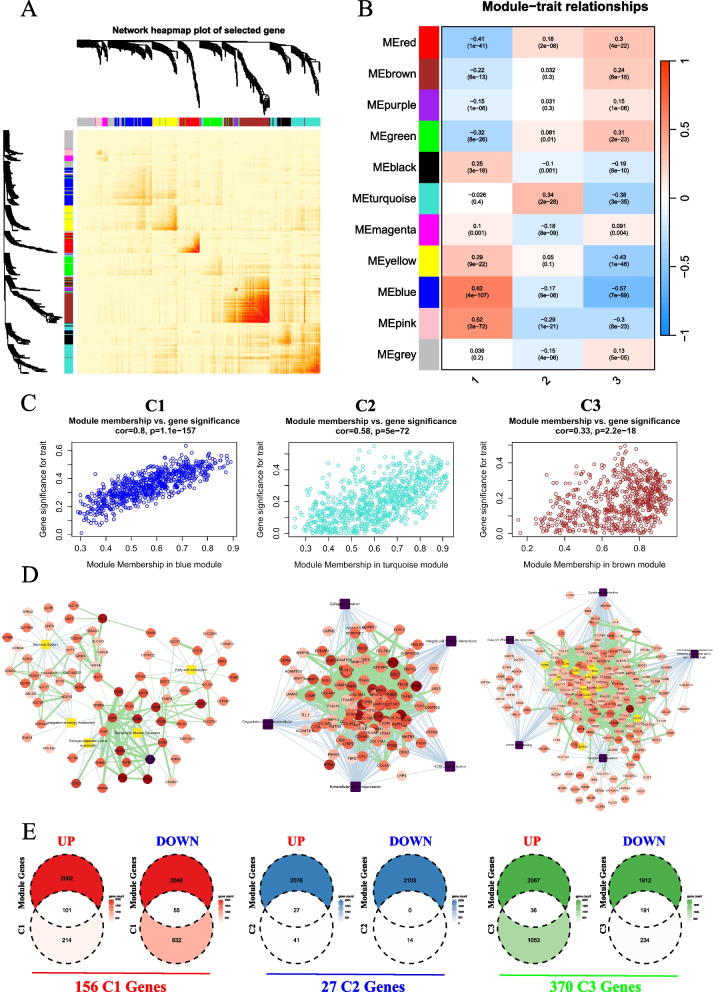
WGCNA analysis associated with the ECM clusters. **A** Heatmap plotting the results of WGCNA analysis based on ECM components- and regulators-related gene expression data identified gene modules with high covariance. **B** Heatmap plotting the module-trait relationships. **C** Dot plot demonstrating the correlation between module membership and gene significance in blue, turquoise, and brown module. **D** Cytoscape illustrating the relative expression level, PPI network, and enriched Reactome pathway of module genes. **E** Venn diagrams showing the intersection of specific upregulated and downregulated DEGs of each ECM cluster with module genes

To delve deeper into the core genes linked to each ECM cluster, we conducted a WGCNA analysis. Outlier samples were excluded from our analysis (Figure S3 A), and a soft threshold of 5 was chosen based on a scale-free R^2 score of 0.85 (Figure S3B). We selected the top 5,000 most variable genes for WGCNA and identified 11 distinct modules (Fig. 3A, and Table S2). Correlation analysis revealed that the blue, turquoise, and brown modules were significantly associated with C1, C2, and C3, respectively (Fig. 3B and C). Specifically, genes in the blue module exhibited higher expression levels in C1, while similar patterns were observed for the turquoise module in C2 and the brown module in C3 (Figure S3 C and S3D).

Next, we explored the interactions among these module genes using PPI analysis, followed by Reactome pathway analysis of the interacting proteins. Consistent with our findings from the DEG analysis (Fig. 1), we discovered that: i) proteins in the blue module were enriched in Neuronal System and Estrogen-dependent Gene Expression pathways; ii) proteins in the turquoise module were associated with ECM remodeling; and iii) proteins in the brown module were enriched in immune-related pathways (Fig. 3D). Subsequently, we filtered specific DEGs from these module genes, resulting in the identification of 156, 27, and 370 specific genes that were significantly correlated with the different ECM clusters (Fig. 3E). These findings highlight various specific genes that are associated with the ECM clusters and may play crucial roles in predicting the outcomes of breast cancer patients.

### Construction of a machine learning model based on ECM cluster-specific DEGs for breast cancer prognosis

To explore the potential of ECM cluster-specific DEGs in predicting breast cancer outcomes, we compiled transcriptome data from TCGA-BRCA and 10 additional independent databases. After rigorous quality control and data integration, we selected four key databases—TCGA, Metabric, SCAN_B, and GEO—to create a meta-cohort comprising 6,736 breast cancer patients for further analysis. This meta-cohort was then divided into a Training cohort (n = 5,392) and a Testing cohort (n = 1,344).

**Fig. 4:**
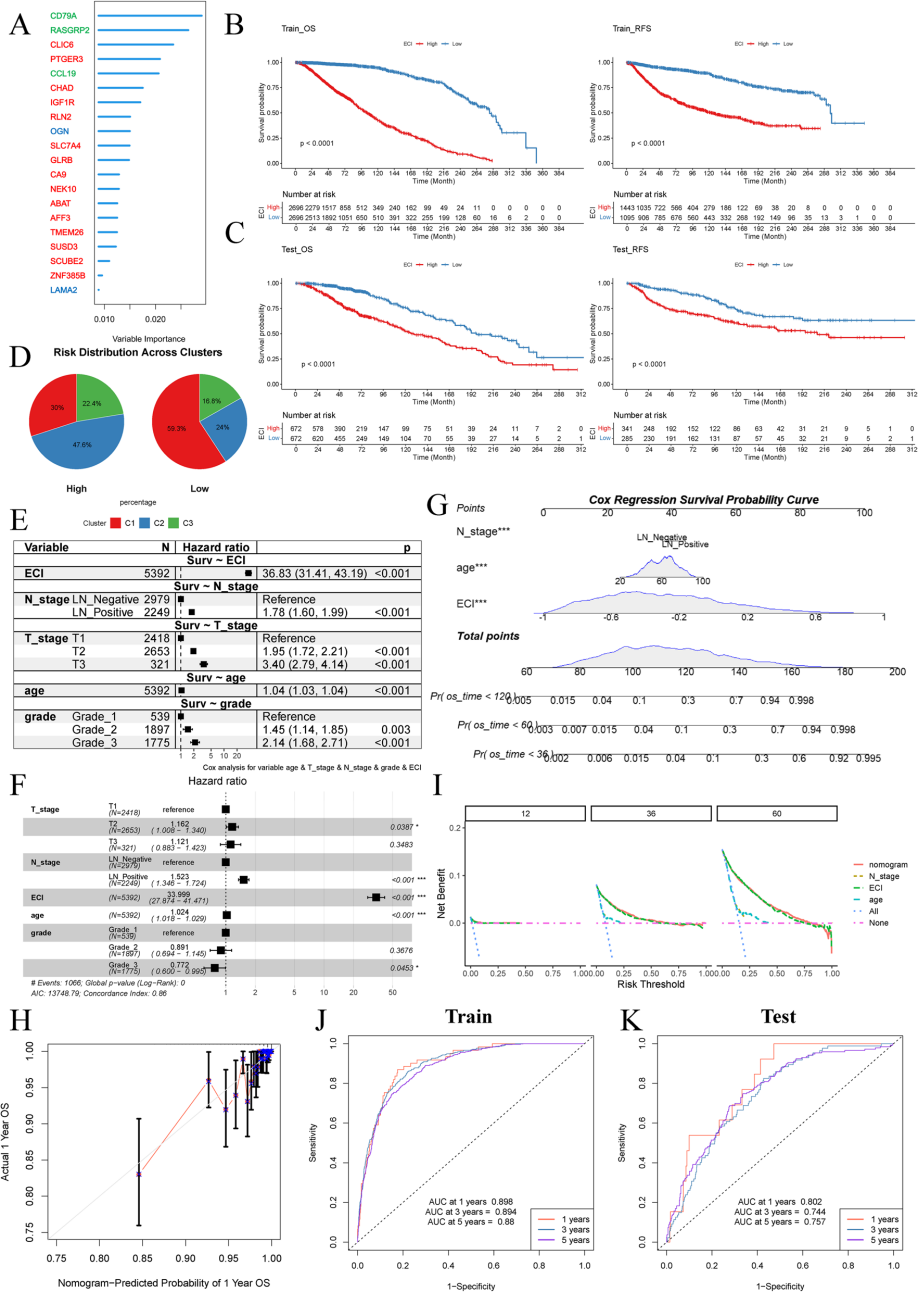
Prognostic model construction and validation. **A** Results of selected top 20 RSF genes according to variable importance. **B** K-M curve plotting OS and RFS status of patients with ECI^High^ and ECI^Low^ in the Training cohort. **C** K-M curve plotting OS and RFS status of patients with ECI^High^ and ECI^Low^ in the Testing cohort. **D** Circle plot illustrating the distribution of each ECM cluster of breast cancer from TCGA-BRCA cohort in ECI^High^ or ECI^Low^ group. **E** Forest plot illustrating results of univariate Cox regression of ECI, N stage, T stage, age and grade in predicting OS of patients in the Training cohort. **F** Forest plot illustrating results of multivariate Cox regression of ECI, N stage, T stage, age and grade in predicting OS of patients in the Training cohort. **G** The nomogram constructed by ECI, N stage, and age demonstrating the accuracy in predicting 1-, 3-, and 5-year OS in the Training cohort. **H** The calibration curves of the nomogram in predicting 1-year OS in the Training cohort. **I** The DCA result of nomogram and individual factors including nomogram, ECI, N stage, and age. **J** ROC curve plotting value of nomogram in predicting OS status of patients in the Training cohort. **K** ROC curve plotting value of nomogram in predicting OS status of patients in the Testing cohort

We employed an integrated machine learning model incorporating 10 distinct algorithms to develop a prognostic model (Liu et al. 2024). The C-index was used to evaluate the performance of various combined algorithms in the Training cohort (Figure S4 A), with RSF emerging as the most effective model for predicting OS. The factors included in this model were identified (Figure S4B). To enhance the model's efficacy, we conducted a second step of RSF Training using a subset of genes and selected the top 20 genes based on their variable importance. A risk score, termed the ECI, was then calculated using these 20 significant genes (Fig. 4A, Table S3). Notably, CD79 A, RAS guanyl releasing protein 2 (RASGRP2), and C–C motif chemokine ligand 19 (CCL19) were specific to C3, osteoglycin (OGN) and laminin subunit alpha 2 (LAMA2) were specific to C2, while the remaining genes were specific to C1.

The ECI was found to be significantly associated with both OS and Relapse-free survival (RFS) in the Training cohort (Fig. 2B). Stratifying patients based on the median ECI revealed a significant correlation between high ECI (ECI^High^) and poor OS and RFS compared to low ECI (ECI^Low^) patients (Fig. 4B, C, S4 C, and S4D). Interestingly, 47.6% of ECI^High^ patients were classified as C2, while 59.3% of ECI^Low^ patients were C1 (Fig. 3A, Table 2). We conducted univariate and multivariate Cox regression analyses in the Training cohort. Our goal was to identify survival-associated factors. These factors included ECI and several clinical variables, such as age, T stage, N stage, and tumor grade. Univariate analysis revealed that ECI, age, T stage, N stage, and grade were all associated with OS (Fig. 4E). Multivariate analysis further confirmed that ECI, age, and N stage were significant factors (*P* < 0.001, Fig. 4F).

**Table 2:** Clinical information of each ECI group in meta data of breast cancer

	Level	ECI^High^	ECI^Low^	P
n		3368	3368	
Subtype (%)	Basal	726 (21.6)	337 (10.0)	< 0.001
	Her2	537 (15.9)	197 (5.8)	
	LumA	931 (27.6)	2085 (61.9)	
	LumB	1011 (30.0)	468 (13.9)	
	Normal	163 (4.8)	281 (8.3)	
T_stage (%)	T1	1158 (34.4)	1856 (55.1)	< 0.001
	T2	1958 (58.1)	1366 (40.6)	
	T3	252 (7.5)	146 (4.3)	
N_stage (%)	LN_Negative	1725 (53.1)	1980 (60.2)	< 0.001
	LN_Positive	1523 (46.9)	1309 (39.8)	
Grade (%)	Grade_1	157 (5.9)	518 (19.9)	< 0.001
	Grade_2	1000 (37.5)	1381 (53.1)	
	Grade_3	1511 (56.6)	703 (27.0)	
Age (%)	< = 35	94 (2.8)	87 (2.6)	< 0.001
	> = 60	2090 (62.1)	1631 (48.4)	
	35 ~ 60	1184 (35.2)	1650 (49.0)	
Chemotherapy (%)	No	1701 (61.2)	1857 (66.0)	< 0.001
	Yes	1080 (38.8)	957 (34.0)	
Hormonal_therapy (%)	No	482 (37.7)	277 (36.6)	0.681
	Yes	798 (62.3)	479 (63.4)	
Menopausal (%)	Post_Menopausal	957 (82.4)	491 (71.9)	< 0.001
	Pre_Menopausal	204 (17.6)	192 (28.1)	

Based on these findings, we constructed a nomogram to predict the OS of breast cancer patients using ECI, age, and N stage (Fig. 4G). The C-index value of the nomogram was 0.861 (95% CI = 0.852–0.872) in the Training cohort and 0.711 (95% CI = 0.678–0.741) in the Testing cohort. Calibration curves demonstrated the accuracy of the nomogram in predicting 1-year survival rates (Fig. 4H). DCA analysis indicated that the ECI performed similarly to the nomogram model but outperformed other predictors used in this study (Fig. 4I). Using the nomogram scores, we observed significant differences in OS between patients with high scores (Score^High^) and those with low scores (Score^Low^) in both the Training (Fig. 4F) and Testing cohorts (Supplementary Fig. 4E-H). The AUC values of the nomogram model for predicting 1-, 3-, and 5-year OS were greater than 0.88 in the Training cohort (Fig. 4J) and 0.74 in the Testing cohort (Fig. 4K). In conclusion, we have successfully established an ECM cluster-specific prognostic model that accurately predicts the outcomes of breast cancer patients in a meta-cohort.

### Patients with C2 or ECI^High^ exabit high infiltration of fibroblasts and low infiltration of immune cells

**Fig. 5:**
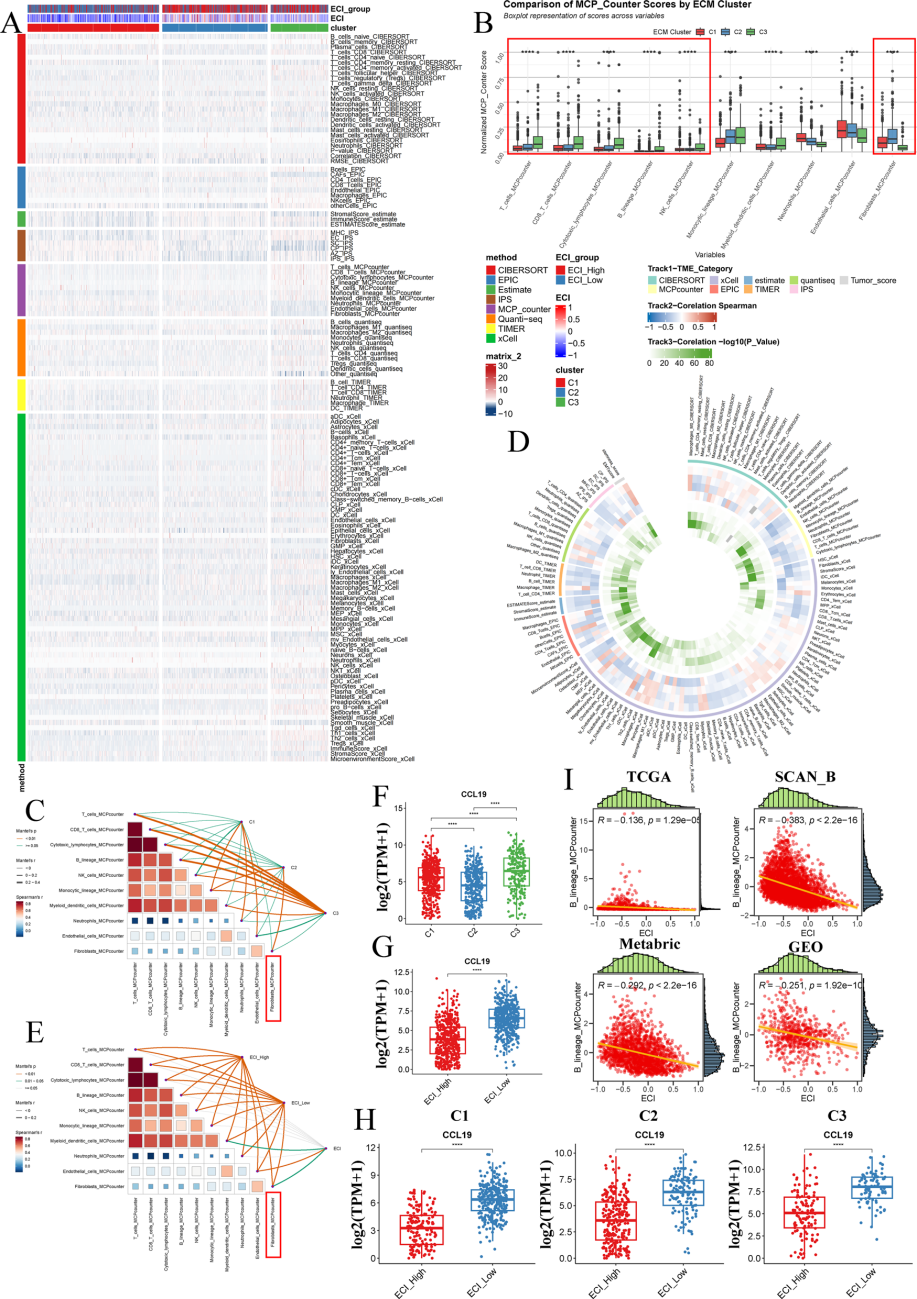
Tumor microenvironment analysis based on ECM clusters or ECI groups. **A** Heatmap plotting results of Cibersort, EPIC, Estimate, IPS, MCP_counter, TIMER, and xCell in the TCGA-BRCA cohort. **B** Box plot illustrating the MCP_counter score of various TME components in each ECM cluster. Wilcox test, *: *P* < 0.05, **: *P* < 0.01, ***: *P* < 0.001, ****: *P* < 0.0001. **C** Heatmap plotting Mantel Test results of each ECM cluster with MCP_Counter score in the TCGA-BRCA cohort. **D** Heatmap plotting results of correlation analysis of ECI with Cibersort, EPIC, Estimate, IPS, MCP_counter, TIMER, xCell and Tumor_score in the TCGA-BRCA, Metabric, SCAN_B, and GEO cohorts. **E** Heatmap plotting Mantel Test results of ECI group or ECI score with MCP_Counter score in the TCGA-BRCA cohort. **F** Box plot illustrating the expression level of CCL19 in each ECM cluster. Wilcox test, *: *P* < 0.05, **: *P* < 0.01, ***: *P* < 0.001, ****: *P* < 0.0001. G Box plot illustrating the expression level of CCL19 in each ECI group. Wilcox test, *: *P* < 0.05, **: *P* < 0.01, ***: *P* < 0.001, ****: *P* < 0.0001. H Box plot illustrating the expression level of CCL19 in each ECI group within various ECM clusters. Wilcox test, *: *P* < 0.05, **: *P* < 0.01, ***: *P* < 0.001, ****: *P* < 0.0001. I Dot plot demonstrating the result of correlation analysis between ECI and B_lineage_MCPconter score in the TCGA-BRCA, Metabric, SCAN_B, and GEO cohorts

To delve into the biological underpinnings of ECI and its association with ECM clusters, we redirected our attention to exploring the relationship among the TME, ECM clusters, and ECI. Consequently, we utilized various deconvolution algorithms, including Cibersort, EPIC, Estimate, IPS, MCP_counter, TIMER, and xCell, to quantify immune infiltration in the TCGA cohort (Fig. 5A). Further analysis with MCP_counter revealed that C3 demonstrates elevated infiltration of multiple immune cells, such as T cells, B cells, and NK cells, compared to other ECM clusters, whereas C2 exhibits a higher infiltration of fibroblasts (Fig. 5B). Mantel Testing analysis further indicated a negative correlation between fibroblast infiltration and the aforementioned immune cells, with C2 exerting a significant influence on fibroblast infiltration (Fig. 5C). Subsequently, we examined the correlation between ECI and TME components across four cohorts (TCGA, SCAN_B, Metabric, and GEO), observing a negative correlation between ECI score and several immune cell types (Fig. 5D). Notably, Mantel Testing analysis also suggested that ECI has a significant impact on fibroblast infiltration (Fig. 5E).

We then assessed the differential expression of ECI genes between ECI^High^ and ECI^Low^ patients, revealing that only carbonic anhydrase 9 (CA9) is overexpressed in the ECI^High^ group, while other genes show higher expression in the ECI^Low^ group (Figure S5 A). Specifically, when examining the expression levels of ECI genes across different ECM clusters (Figure S5B). And we found that CCL19 is downregulated in C2 compared to other ECM clusters (Fig. 5G). CCL19 also exhibited lower expression in the ECI^High^ group across all patients (Fig. 5H) and within each ECM cluster (Fig. 5I). Interestingly, ECI demonstrated a significant negative correlation with B cell infiltration across all four cohorts (Fig. 5J). Given that CD79 A, one of the ECI genes with the highest variable importance, is a B cell marker, we hypothesize that fibroblasts and B cells may play crucial roles in this context.

### Single-cell transcriptome analysis identifies CFD^+^ and ANGPTL4^+^ fibroblasts as key regulators of b cells in breast cancer

**Fig. 6:**
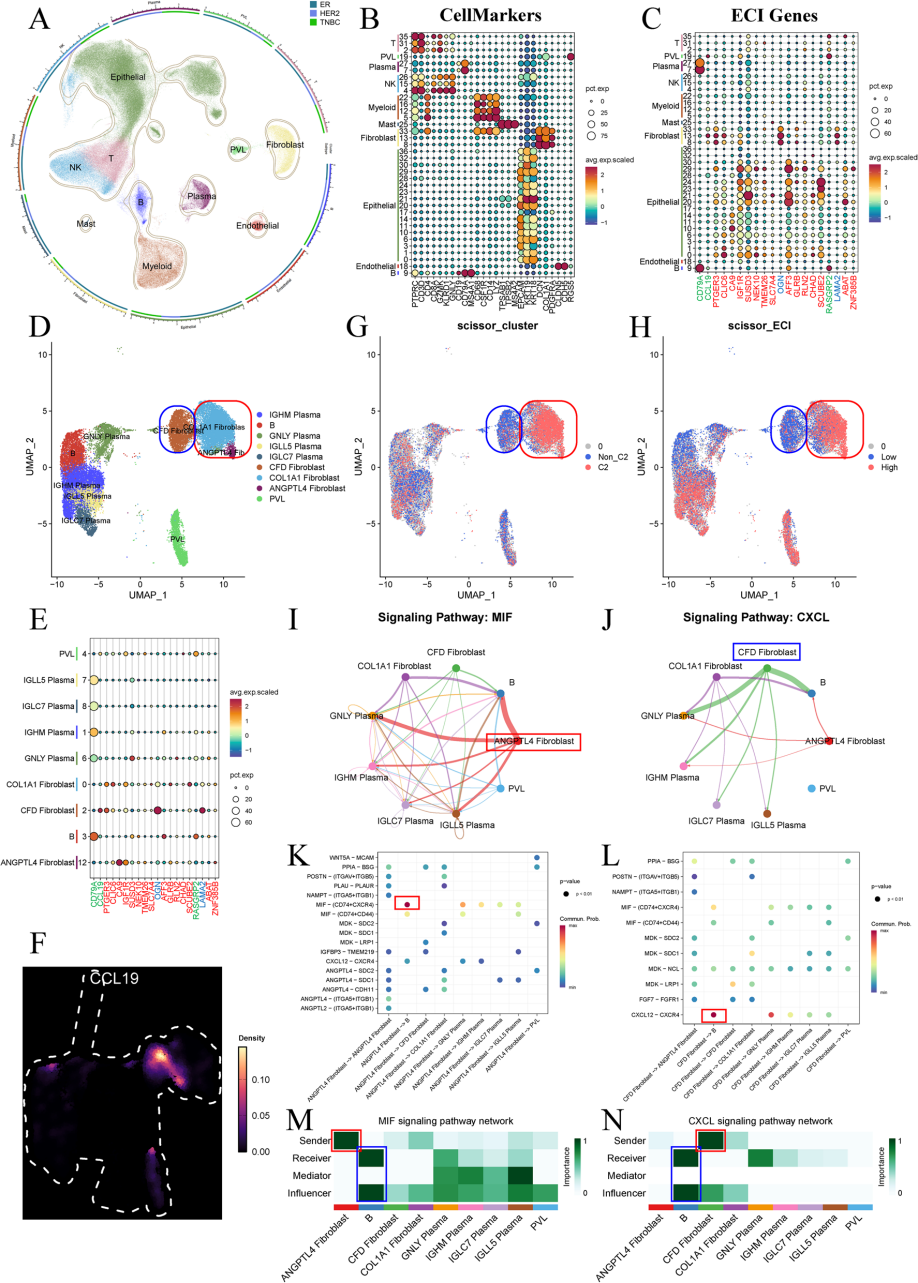
scRNA-seq analysis in breast cancer patients. **A** UMAP plot showing 10 celltypes from 31 breast cancer patients and outlier lines demonstrating the percentage of each celltype in ER, HER2 or TNBC. **B** Dot plot demonstrating the expression level of cell markers in each cell cluster and celltype. **C** Dot plot demonstrating the expression level of ECI genes in each cell cluster and celltype. **D** UMAP plot showing 9 celltypes after re-clustering of PVL, fibroblasts, and B-cell lineage. **E** Dot plot demonstrating the expression level of ECI genes in each cell cluster and celltype of PVL, fibroblasts, and B-cell lineage. **F** Density plot of CCL19 expression in each cell of PVL, fibroblasts, and B-cell lineage. **G** UMAP plot showing phenotype of each cell linking ECM clusters based on the bulk RNA-seq data of TCGA-BRCA. **H** UMAP plot showing phenotype of each cell linking ECI group based on the bulk RNA-seq data of TCGA-BRCA. **I** Ligand-receptor interaction analysis of MIF pathway among PVL, fibroblasts, and B-cell lineage. **J** Ligand-receptor interaction analysis of CXCL pathway among PVL, fibroblasts, and B-cell lineage. **K** Dot plot demonstrating different MIF signaling pathways among PVL, fibroblasts, and B-cell lineage. **L** Dot plot demonstrating different CXCL signaling pathways among PVL, fibroblasts, and B-cell lineage. **M** Heatmap plotting cell interaction of MIF signaling pathway network among PVL, fibroblasts, and B-cell lineage. **N** Heatmap illustrating cell interaction of CXCL signaling pathway network among PVL, fibroblasts, and B-cell lineage

To explore the expression of core ECI genes in specific cell types, we analyzed scRNA-seq data from the GSE161529 database. After stringent quality control and data integration, we retained 210,293 cells from 31 breast cancer patients for further analysis. Our analysis revealed 36 distinct cell clusters across ER, TNBC, and HER2 patient samples (Figures S6 A, S6B, and Table S4). Following the exclusion of low-quality clusters, such as cluster 34, we annotated the remaining cells into ten cell types: epithelial cells, T cells, NK cells, B cells, plasma cells, myeloid cells, mast cells, endothelial cells, fibroblasts, and perivascular-like (PVL), based on the CellMarker 2.0 website (Fig. 6A). The expression levels of cell markers for each cell type are shown in Fig. 6B. Notably, we found that CD79 A and RASGRP2, identified as C3-specific genes, are expressed in B cells and plasma cells (Figure S6 C). Additionally, the C3-specific gene CCL19, along with the C2-specific genes OGN and LAMA2, showed high expression levels in fibroblasts and PVL (Figure S6 C). These findings, consistent with our previous bulk RNA sequencing results, support the involvement of fibroblasts and B cells in ECM-driven breast cancer progression.

Consequently, we selected fibroblasts, B cells, and PVL for further subcluster analysis. After re-clustering (Figure S7 A, and Table S5), we identified specific subclusters of B cells and fibroblasts, including: i) five B lineage clusters (B cells, IGHM plasma cells, GNLY plasma cells, IGLL5 plasma cells, and IGLC7 plasma cells), ii) three fibroblast clusters (COL1 A1 fibroblasts, CFD fibroblasts, and ANGPTL4 fibroblasts), and iii) PVL (Fig. 6D). We observed that the only risk factor, CA9, is highly expressed in ANGPTL4 fibroblasts, while protective ECI genes are expressed in other cell types (Fig. 6E). Specifically, CFD fibroblasts exclusively express CCL19, which was previously found to be negatively associated with C2 (Fig. 6F).

Next, we employed the Scissor algorithm to further investigate the relationships among ECM clusters, ECI, and cell types (Sun et al. 2022). Using bulk transcriptome results from the TCGA cohort, we identified cells with similar expression patterns to ECM clusters (Fig. 6G) or ECI groups (Fig. 6H). As anticipated, CFD fibroblasts exhibited a phenotype distinct from C2 (Non_C2) and ECI^Low^, while COL1 A1 fibroblasts and ANGPTL4 fibroblasts were associated with the C2 and ECI^High^ phenotypes (Fig. 6G and H). We then analyzed ligand-receptor interactions among these cell types, revealing multiple interaction pathways (Figure S7B). Our analysis showed that ANGPTL4 fibroblasts regulate B cell lineage via the MIF pathway (Fig. 6I), while CFD fibroblasts influence B cell lineage through the CXCL pathway (Fig. 6J). Specifically, ANGPTL4 fibroblasts and CFD fibroblasts had the great impact on B cells (Figs. 6K and L), and these fibroblasts were identified as the senders of the MIF or CXCL signaling pathways, with B cells and other plasma cells serving as the primary receivers and influencers (Fig. 6M and N). Overall, these findings suggest that the crosstalk between fibroblasts and B cell lineage plays a significant role in ECM-driven breast cancer progression.

### Investigating the roles of CFD and ANGPTL4 in the outcomes of cancer patients undergoing immunotherapy

**Fig. 7:**
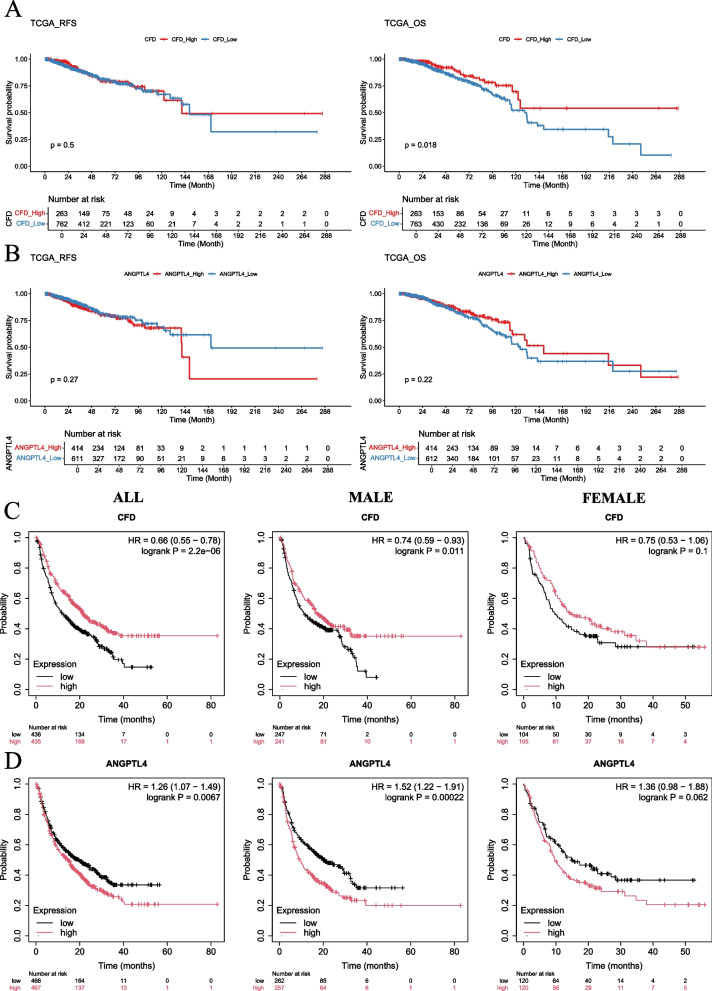
Investigating the role of CFD and ANGPTL4 in influencing survival status in breast cancer and cancer patients received immunotherapy. **A** K-M curve plotting OS and RFS status of patients with CFD^High^ and CFD^Low^ in the TCGA-BRCA cohort. **B** K-M curve plotting OS and RFS status of patients with ANGPTL4^High^ and ANGPTL4^Low^ in the TCGA-BRCA cohort. **C** K-M curve plotting OS status of CFD^High^ and CFD^Low^ cancer patients treated with immunotherapy via using KM-Plotter database. **D** K-M curve plotting OS status of ANGPTL4^High^ and ANGPTL4^Low^ cancer patients treated with immunotherapy via using KM-Plotter database

Ultimately, our objective was to examine the correlation between fibroblast cell markers, notably CFD and ANGPTL4, and the prognosis of breast cancer patients. Within the TCGA cohort, we discovered a significant association between CFD and improved OS in breast cancer patients (Fig. 7A). Additionally, we noted a trend suggesting that elevated ANGPTL4 expression might positively influence the OS of breast cancer patients (Fig. 7B). Given the reported involvement of fibroblasts and B-cell lineage in the response to immunotherapy, we further explored the relationship between CFD and ANGPTL4 with immunotherapy response using the KM-Plotter database (Pei et al. 2023; Engelhard et al. 2021). Strikingly, our findings revealed that CFD enhances the efficacy of immunotherapy in both male and female patients (Fig. 7C), whereas ANGPTL4 appears to detract from the therapeutic outcomes of patients receiving immunotherapy (Fig. 7D).

## Discussion

The ECM is composed of a variety of structural proteins and small molecules, some of which undergo post-translational modifications catalyzed by enzymes in the tumor microenvironment (TME). Our study utilized ECM components and regulators to develop a prognostic model, which demonstrated an AUC of 0.861 (95% CI = 0.852–0.872) in the training cohort and 0.711 (95% CI = 0.678–0.741) in the testing cohort (Fig. 4E-K) within a breast cancer meta-dataset. Several other studies have also explored the impact of ECM components on cancer patient outcomes. For instance, Amelia et al. created a model based on ECM signatures to distinguish the squamous subtype of non-small cell lung cancer from normal tissues and found that patients with high ECM expression had poorer outcomes (Parker et al. 2022). Deng et al. developed a prognostic model based on ECM signatures in 122 gastric cancer patient-derived tumor xenografts, achieving an AUC of 0.75 in the training cohort and 0.60 in the testing cohort for 5-year survival (Deng et al. 2023). Additionally, Wei et al. examined the role of metastasis and basement membrane-related genes in predicting outcomes for hepatocellular carcinoma, reporting an AUC of 0.706 in the training cohort and 0.683 in the testing cohort for 3-year survival (Wei et al. 2024). In comparison to these studies, our model exhibits reliable effectiveness in predicting long-term survival outcomes in breast cancer, potentially due to the integration of ECM components with regulators. Another plausible explanation is that the ECM may play a more significant role in influencing outcomes for breast cancer patients compared to other cancer types.

Numerous studies have reported that specific ECM components can regulate cancer phenotypes by stimulating signaling pathways. For example, heterogeneous nuclear ribonucleoprotein C (HNRNPC) promotes discoidin domain receptor tyrosine kinase 1 (DDR1) transcription by recognizing Vir like M6 A methyltransferase associated (VIRMA)-exposed m6 A modifications on transcription factor AP-2 alpha (TFAP2 A) mRNA, leading to enhanced collagen fiber alignment. This, in turn, reduces the infiltration of anti-tumor immune cells and facilitates immune escape in breast cancer (Lian et al. 2023). Additionally, laminin subunit gamma 2 (LAMC2) targets zinc finger E-Box binding homeobox 1 (ZEB1) through activation of the CD44/STAT3 signaling pathway, promoting the proliferation and migration of TNBC cells, suggesting its potential as a therapeutic target for TNBC patients (Wang et al. 2024). Beyond breast cancer, ECM components like COL1 A1 have also been shown to affect the infiltration and function of immune cells, contributing to immunotherapy resistance in pancreatic cancer (Chen et al. 2022a, 2021). In our study, fibroblasts expressing COL1 A1 were associated with C2 and ECI^High^ phenotypes (Fig. 6D, H, and I). However, we focused our attention on CFD^+^ and ANGPTL4^+^ due to their potential regulatory roles in B-cell lineage (Fig. 6I-N). C2 phenotype was recognized with the enrichment of ECM components and regulators (Fig. 1A and G). Whereas CFD is correlated with Non_C2 phenotype and optimal outcome of breast cancer patients. These results indicated that CFD and ANGPTL4 may participate in the remodeling of ECM or response to the ECM remodeling.

CFD has been reported to be associated with the function of fibroblasts. For example, a prior study revealed that knocking down CFD in senescent fibroblasts notably decreased the elevation of matrix metallopeptidase 1 (MMP1) in co-cultured young fibroblasts (Ezure et al. 2019). Additionally, senescent cells adversely affect matrix production and contribute to the degradation of neighboring fibroblasts in the dermal layer, partly through the secretion of CFD (Ezure et al. 2019). Another study identified CFD as the most dependable predictive biomarker for successful tendon healing through proteomic profiling (Chen et al. 2022b). Further bioinformatic and experimental investigations, utilizing both inflammatory and proliferative fibroblast models, have shown that CFD potentially enhances repair by regulating cell migration and modulating the expression of COL1 A1 in dense connective tissues (Chen et al. 2023). These works highlighted that CFD is a crucial gene that governs the generation of proteins and enzyme to guarantee the healthy of ECM, which also contributed to the tissue healing. Thus, fibroblasts expressing CFD, which identified in our work, may play a role in regulating ECM into a healthier status.

ANGPTL4, a notably significant marker, has been extensively reported to be associated with fibroblast function and cancer progression. In TNBC, STAT3 activation in fibroblasts stimulates the expression of ANGPTL4 and other factors, thereby exerting a pro-tumorigenic effect (Avalle et al. 2022). Furthermore, a study utilizing scRNA-seq revealed that fibroblasts in the heart may interact with endothelial cell lineages through the over-secretion of ANGPTL4, exhibiting an anti-angiogenic function (Li et al. 2024b). Recent research indicates that the suppression of Angptl4 results in a dose-dependent elimination of epithelial-mesenchymal transition (EMT)-mediated chemoresistance and tumor self-organization in three-dimensional (3D) cultures, ultimately leading to reduced metastatic potential and impaired growth of tumor xenografts (Liao et al. 2024), which consistent with our findings that C2 phenotype showed higher enrichment of EMT (Figure S1 F). These findings strongly imply that ANGPTL4 may serve as a key factor in sensing the ECM to regulate the phenotype of cells, encompassing both fibroblasts and cancer cells. Though we found that CFD and ANGPTL4 were significantly associated with the response to immunotherapy, the roles of them in immunotherapy is still unclear.

We also identified CCL19 as a gene specifically downregulated in the C2 phenotype (Fig. 5F-H), with significant expression observed in fibroblasts, particularly in CFD fibroblasts (Fig. 6C and F). Our findings align with existing research. For example, fibroblasts producing CCL19 have been shown to promote the formation of tertiary lymphoid structures, thereby enhancing the anti-tumor IgG response in colorectal cancer liver metastasis (Zhang et al. 2024). Additionally, lung cancer contains spatially organized stem-immunity hubs that are distinct from mature tertiary lymphoid structures and are enriched with stem-like TCF7^+^PD-1^+^CD8^+^ T cells, activated CCR7^+^LAMP3^+^ dendritic cells, CCL19^+^ fibroblasts, and chemokines that organize these cells, all of which are associated with a response to immunotherapy (Chen et al. 2024). Notably, a study has identified that CCL19-expressing fibroblastic reticular cells (FRCs) generate interconnected T cell environments (TEs) in human non-small cell lung cancer, including tertiary lymphoid structures and T cell tracks (Onder et al. 2024). The ablation of intratumoral FRC precursors was found to decrease antitumor T cell activity, resulting in reduced tumor control during coronavirus vector-based immunotherapy (Onder et al. 2024). Based on these findings, we hypothesize that breast cancer patients with the C2 phenotype exhibit low expression of CCL19, contributing to the creation of an immunosuppressive microenvironment and subsequently leading to poor patient outcomes. Consequently, targeting these fibroblasts may represent an optimal strategy to improve patient prognosis.

## Conclusion

We successfully identified distinct ECM clusters in breast cancer patients, which were closely associated with patient survival outcomes. Based on these ECM clusters, we constructed an effective prognostic model and identified that high expression of ANGPTL4 may impair immunotherapy efficacy, whereas CFD shows potential to enhance immunotherapy response. Therefore, targeted intervention strategies against ANGPTL4 and CFD could emerge as novel approaches to improve breast cancer immunotherapy. For instance, specific antibodies or small molecule inhibitors could be designed to block ANGPTL4 function, or CFD agonists could be utilized to bolster patients'immune responses. In summary, our study not only presents novel biomarkers for predicting breast cancer prognosis but also opens up new avenues for breast cancer immunotherapy. Future research should further elucidate the specific mechanisms of ANGPTL4 and CFD in breast cancer and develop clinical detection methods and treatment strategies based on these biomarkers, thereby advancing precision medicine in breast cancer.

## Supplementary Information

Supplementary Material 1: Figure S1.Results of NMF cluster rank analysis.Volcano plot illustrating DEGs among each ECM clusters.Venn diagrams showing the intersection of downregulated DEGs obtained from the comparation among each ECM clusters.Box plot illustrating the top 5 enriched Reactome pathways of specific downregulated DEGs in each ECM cluster.Box plot illustrating the enrichment status of HALLMARK gene sets in C1, C2, C3. Wilcox test, *: *P* < 0.05, **: *P* < 0.01, ***: *P* < 0.001, ****: *P* < 0.0001

Supplementary Material 2: Figure S2. A Oncoplot demonstrating the results of gene mutation in each ECM clusters. B Box plot illustrating the top 20 mutant genes in each ECM clusters. C Heatmap plotting co-occurrence and mutually exclusive gene mutation status in each ECM clusters

Supplementary Material 3: Figure S3. A Forest plot illustrating the cluster of samples base on the top 5,000 variable genes in TCGA-BRCA cohort. B Results of soft-threshold power of WGCNA analysis. C Box plot illustrating the difference of module genes in each ECM clusters. Kruskal-Wallis test. D Heatmap plotting expression level of blue, turquoise, and brown genes in each ECM clusters

Supplementary Material 4: Figure S4. A Heatmap plotting the results of CDI of various prognostic models. B RSF model selected from prognostic models. C Triplet graph demonstrating the distribution of patients, alive status, and expression level of ECI genes in the Training cohort. D Triplet graph demonstrating the distribution of patients, alive status, and expression level of ECI genes in the Testing cohort. E K-M curve plotting OS status of patients with NomogramHigh and NomogramLow in the Training cohort. F K-M curve plotting RFS status of patients with NomogramHigh and NomogramLow in the Training cohort. G K-M curve plotting OS status of patients with NomogramHigh and NomogramLow in the Testing cohort. H K-M curve plotting RFS status of patients with NomogramHigh and NomogramLow in the Testing cohort

Supplementary Material 5: Figure S5. A Box plot illustrating the expression level of ECI genes in each ECI group. Wilcox test, *: *P* < 0.05, **: *P* < 0.01, ***: *P* < 0.001, ****: *P* < 0.0001. B Box plot illustrating the expression level of ECI genes in each ECM cluster. Wilcox test, *: *P* < 0.05, **: *P* < 0.01, ***: *P* < 0.001, ****: *P* < 0.0001

Supplementary Material 6: Figure S6. A UMAP plot showing 36 cell clusters from 31 breast cancer patients. B UMAP plot showing cells derived from ER, HER2 or TNBC patients. Density plot of expression level of ECI genes in each cell

Supplementary Material 7: Figure S7. A Heatmap plotting expression level of top 5 cell markers in each cell cluster of PVL, fibroblasts, and B-cell lineage. B Ligand-receptor interaction analysis among PVL, fibroblasts, and B-cell lineage

Supplementary Material 8.

## Data Availability

No datasets were generated or analysed during the current study.
