# Supplementary figures and images for: Transcriptome-wide analysis reveals potential roles of CFD and ANGPTL4 in fibroblasts regulating B cell lineage for extracellular matrix-driven clustering and novel avenues for immunotherapy in breast cancer
Source: Mol Med. 2025 May 8;31:179. doi: 10.1186/s10020-025-01237-y (PMC12063413; doi:10.1186/s10020-025-01237-y)

## A NMF rank survey

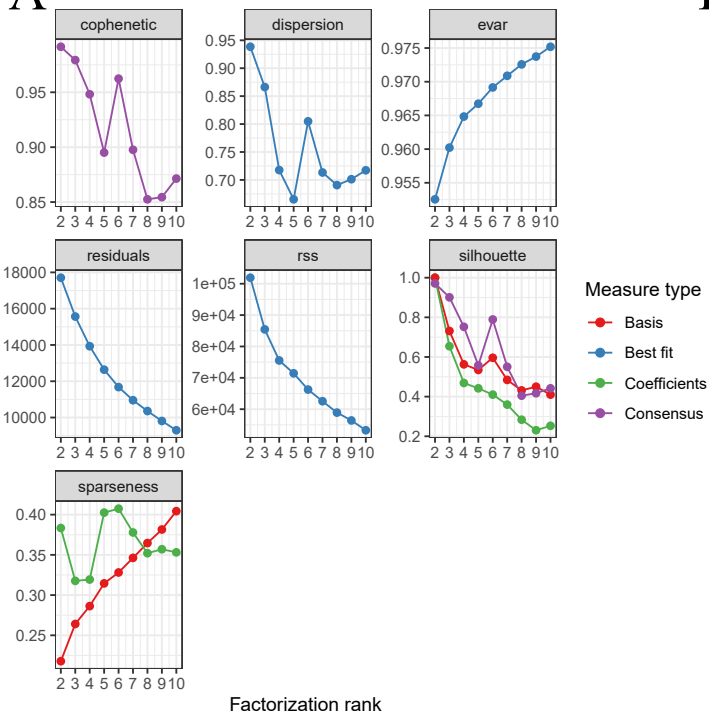

## B

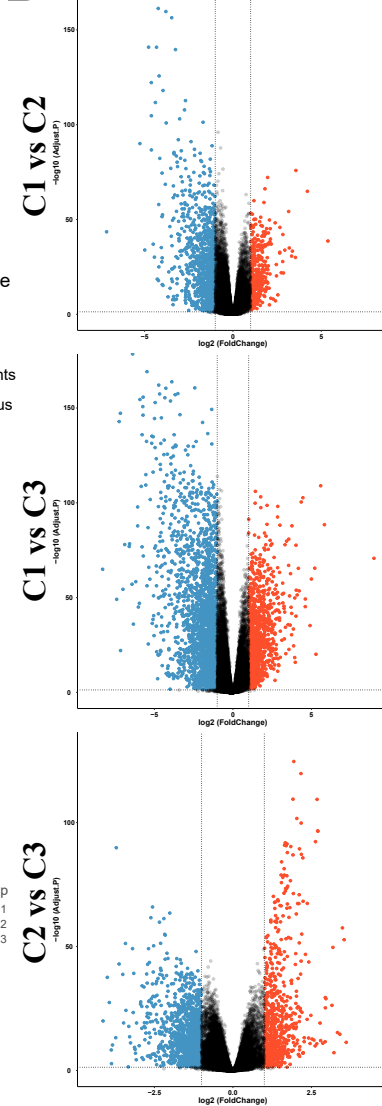

## C

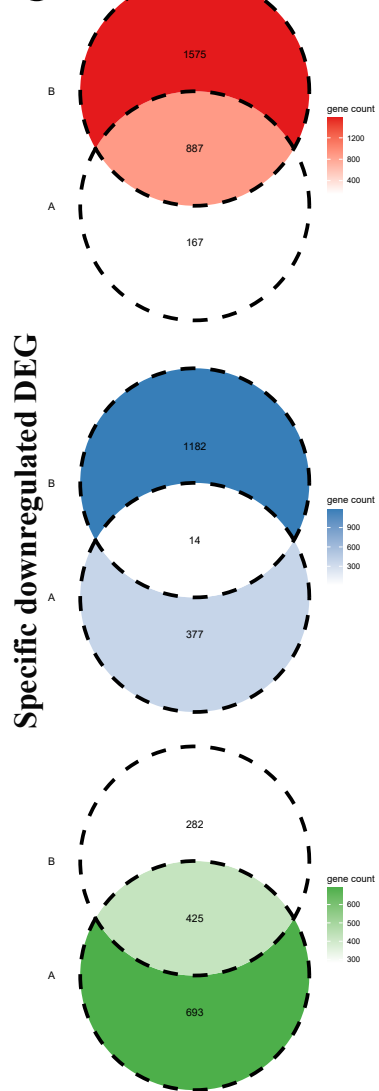

## D

### Specific downregulated DEG

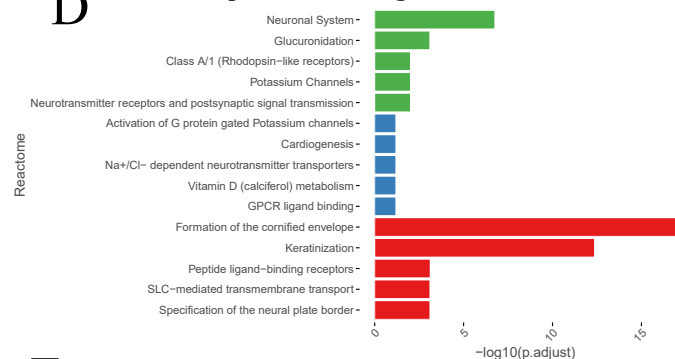

## E

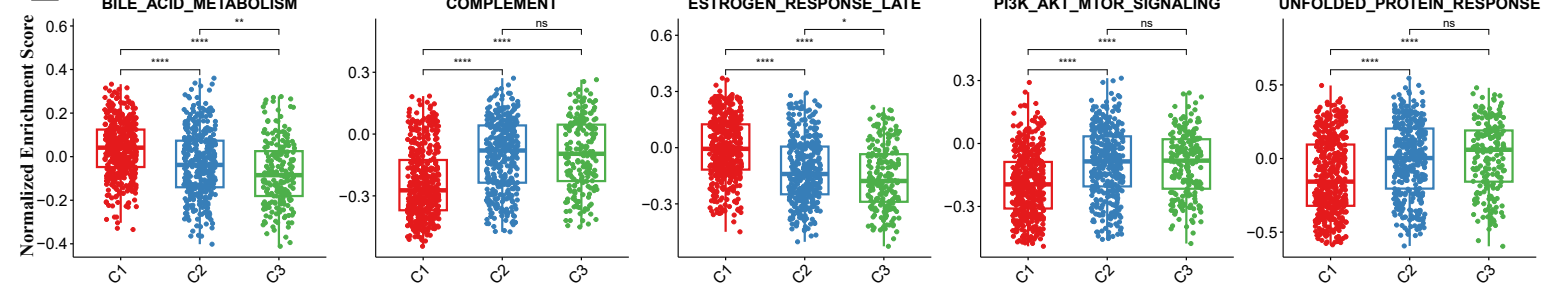

## F

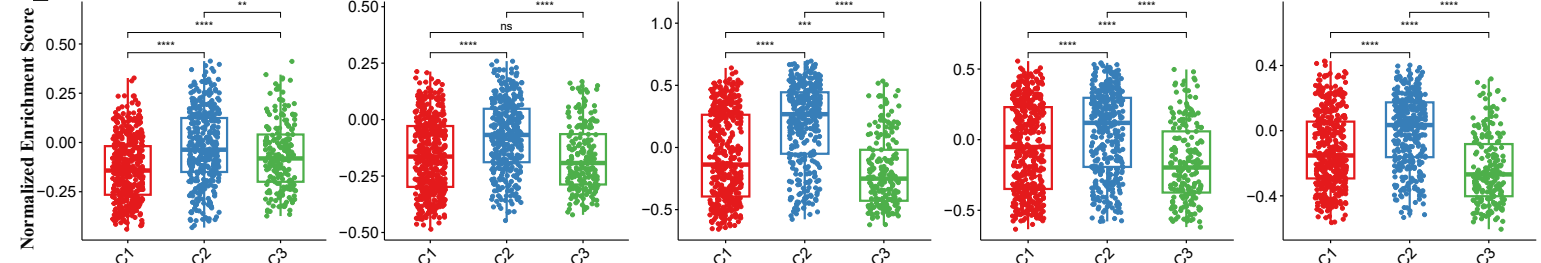

## G

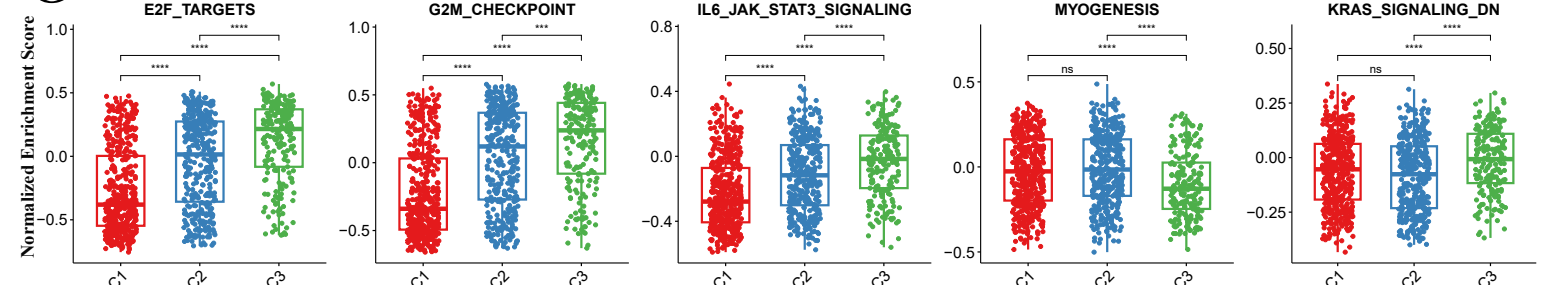

Supplement: Supplementary file 1 — Supplementary Material 1: Figure S1.Results of NMF cluster rank analysis.Volcano plot illustrating DEGs among each ECM clusters.Venn diagrams showing the intersection of downregulated DEGs obtained from the comparation among each ECM clusters.Box plot illustrating the top 5 enriched Reactome pathways of specific downregulated DEGs in each ECM cluster.Box plot illustrating the enrichment status of HALLMARK gene sets in C1, C2, C3. Wilcox test, *: P < 0.05, **: P < 0.01, ***: P < 0.001, ****: P < 0.0001 [file 10020_2025_1237_MOESM1_ESM.pdf]

C3

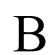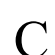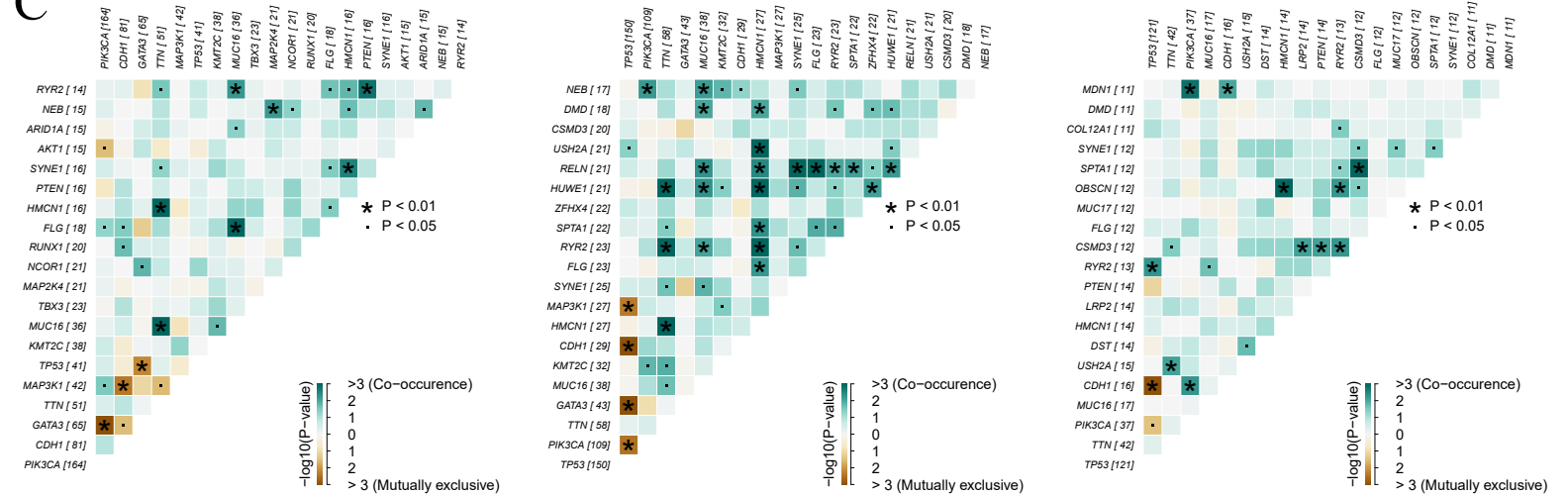

Supplement: Supplementary file 2 — Supplementary Material 2: Figure S2. A Oncoplot demonstrating the results of gene mutation in each ECM clusters. B Box plot illustrating the top 20 mutant genes in each ECM clusters. C Heatmap plotting co-occurrence and mutually exclusive gene mutation status in each ECM clusters [file 10020_2025_1237_MOESM2_ESM.pdf]

A

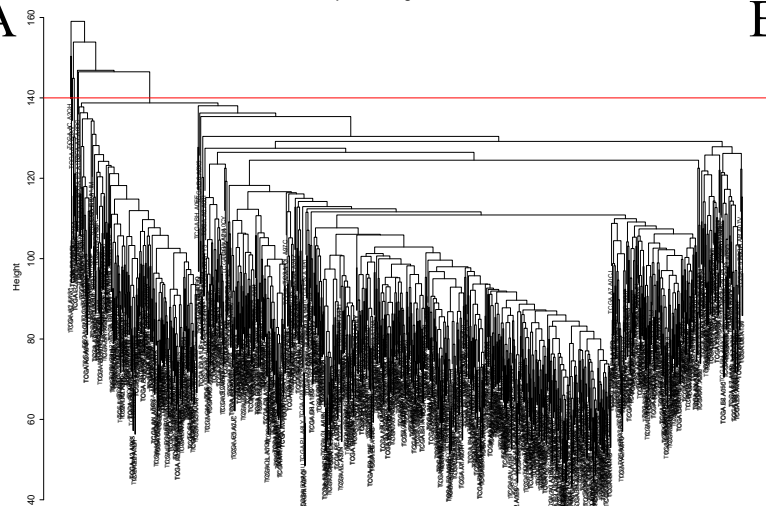

B

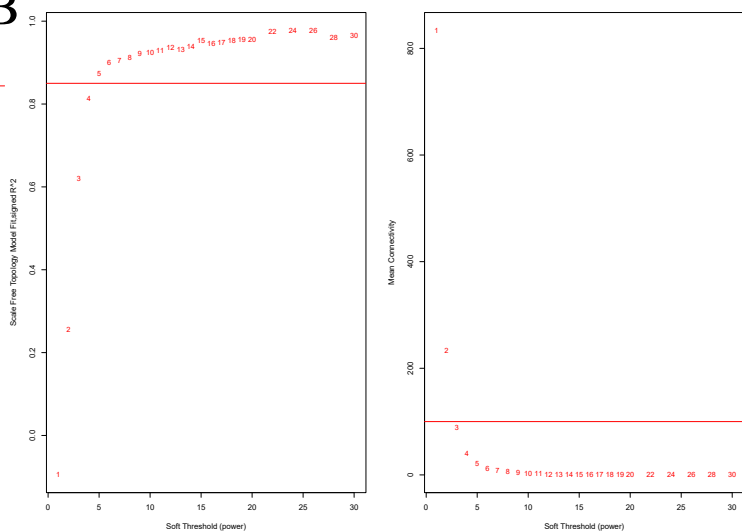

C

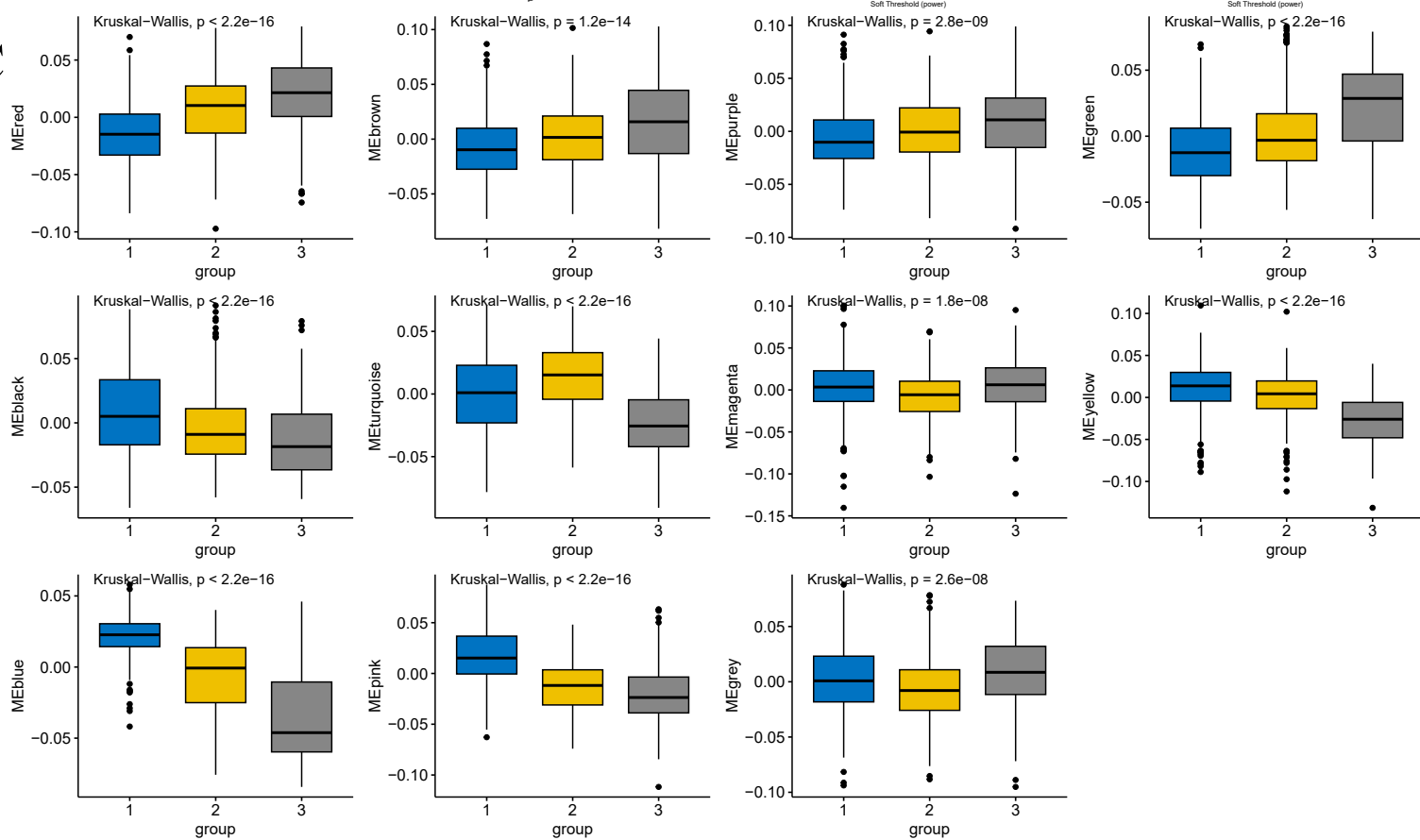

D

Blue

Turquoise

Brown

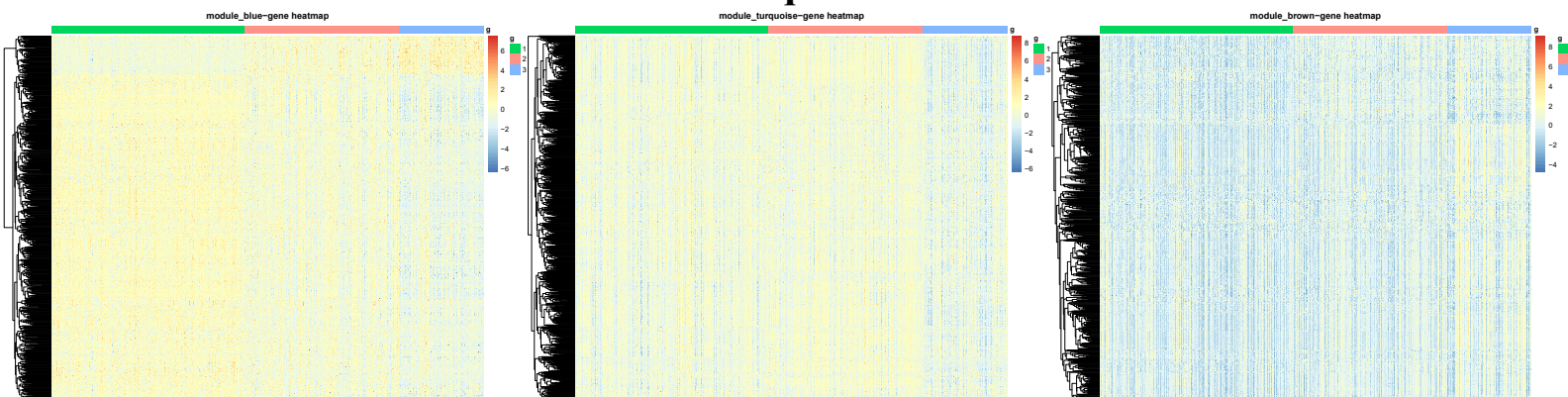

Supplement: Supplementary file 3 — Supplementary Material 3: Figure S3. A Forest plot illustrating the cluster of samples base on the top 5,000 variable genes in TCGA-BRCA cohort. B Results of soft-threshold power of WGCNA analysis. C Box plot illustrating the difference of module genes in each ECM clusters. Kruskal-Wallis test. D Heatmap plotting expression level of blue, turquoise, and brown genes in each ECM clusters [file 10020_2025_1237_MOESM3_ESM.pdf]

A

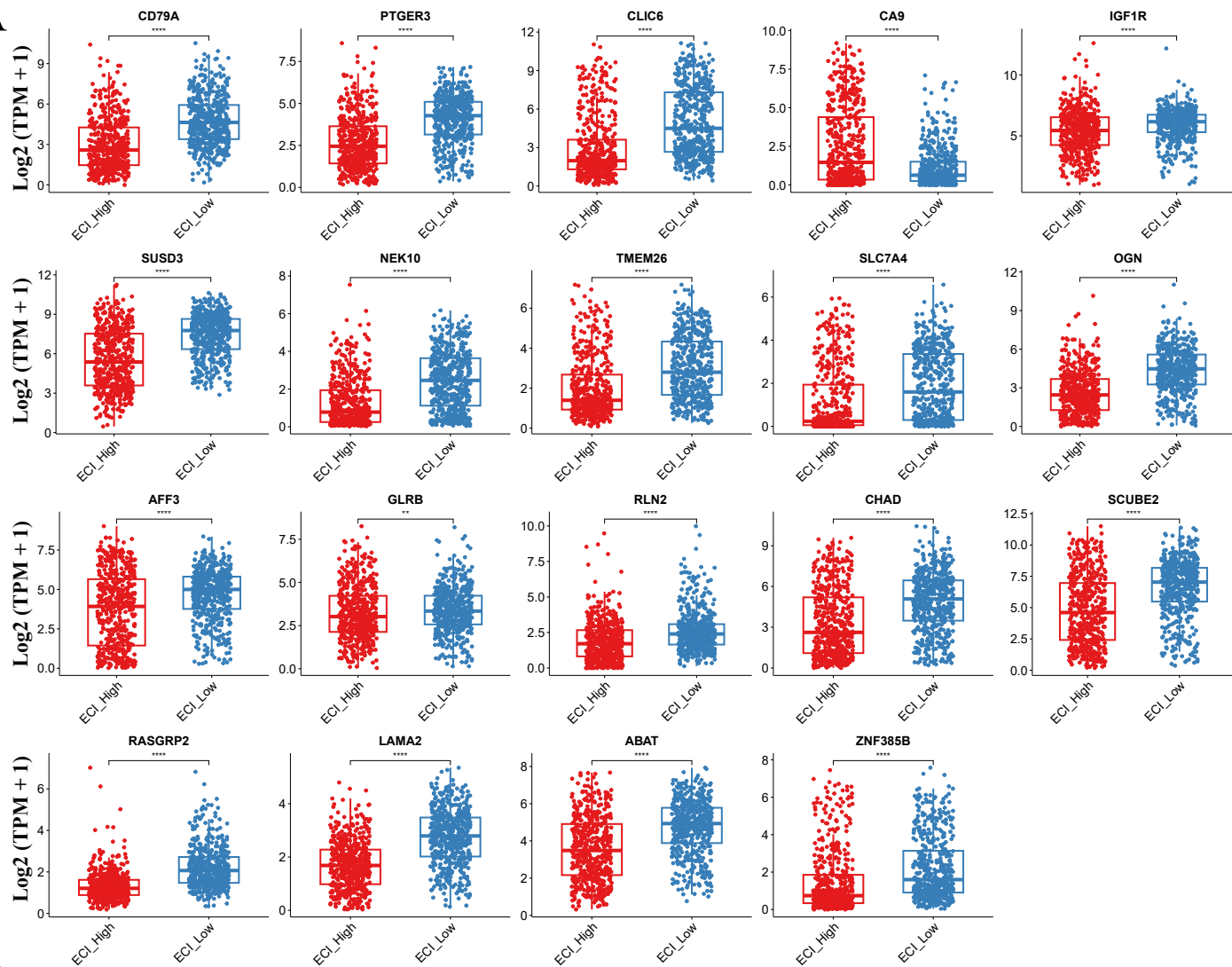

B

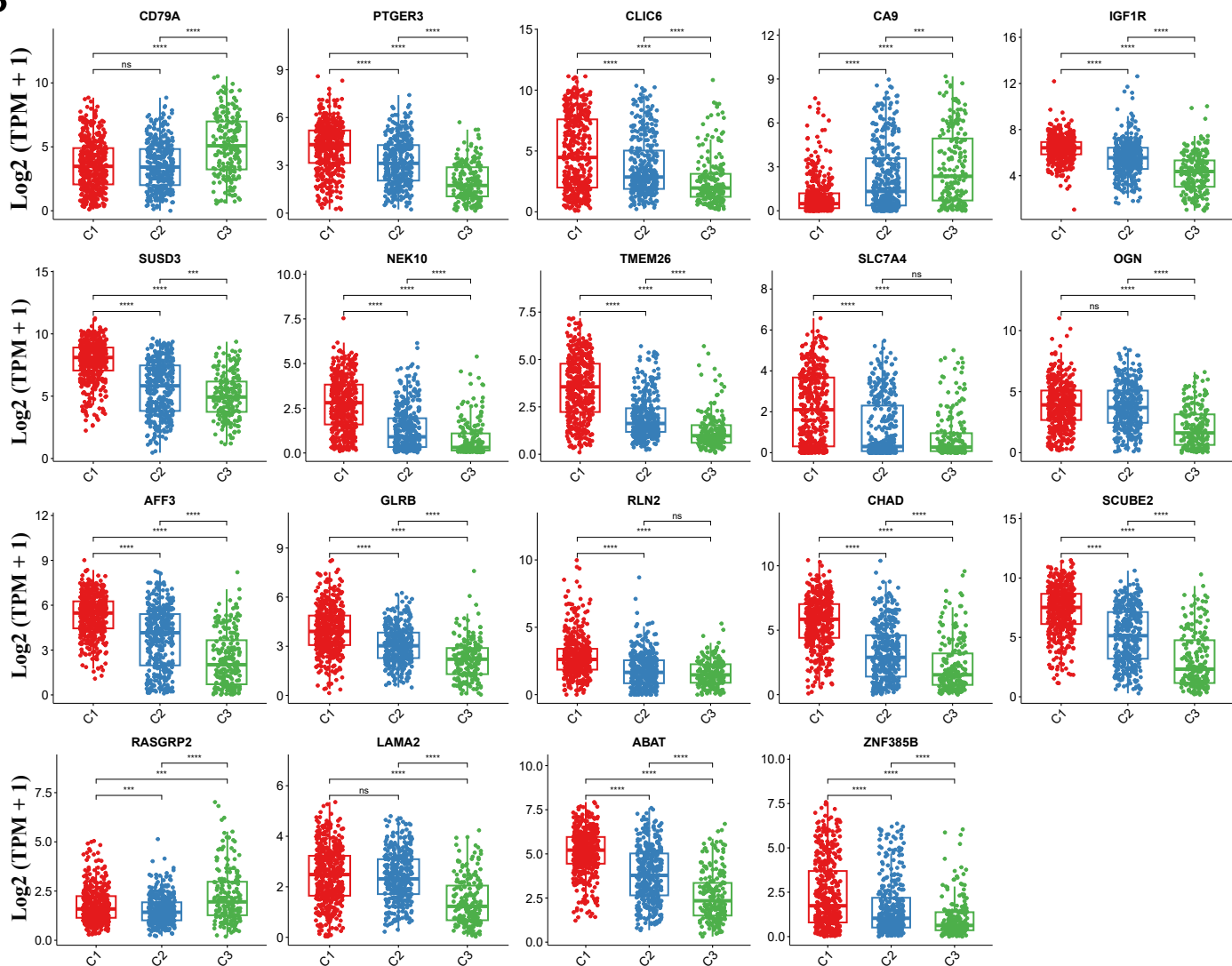

Supplement: Supplementary file 5 — Supplementary Material 5: Figure S5. A Box plot illustrating the expression level of ECI genes in each ECI group. Wilcox test, *: P < 0.05, **: P < 0.01, ***: P < 0.001, ****: P < 0.0001. B Box plot illustrating the expression level of ECI genes in each ECM cluster. Wilcox test, *: P < 0.05, **: P < 0.01, ***: P < 0.001, ****: P < 0.0001 [file 10020_2025_1237_MOESM5_ESM.pdf]

A

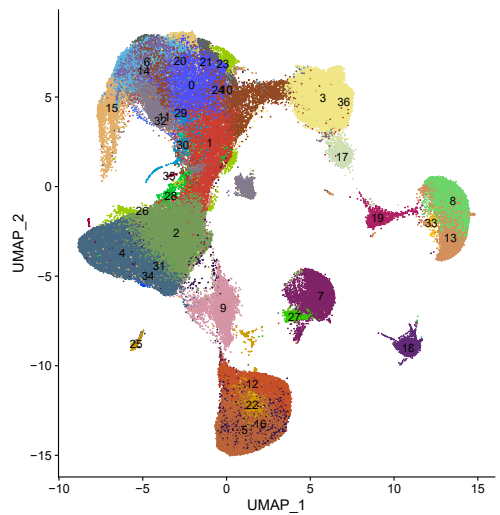

B

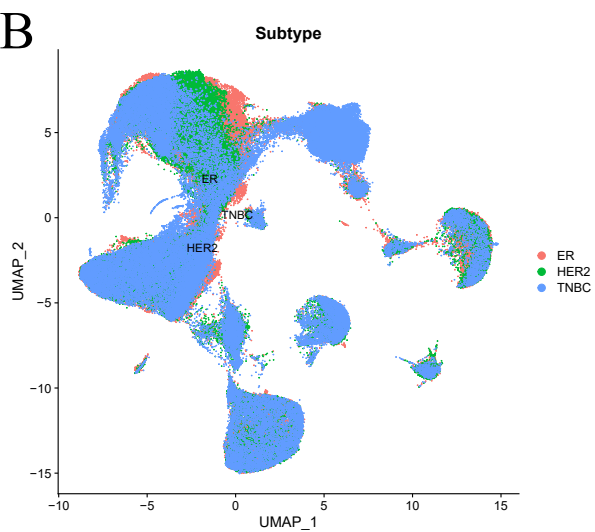

C

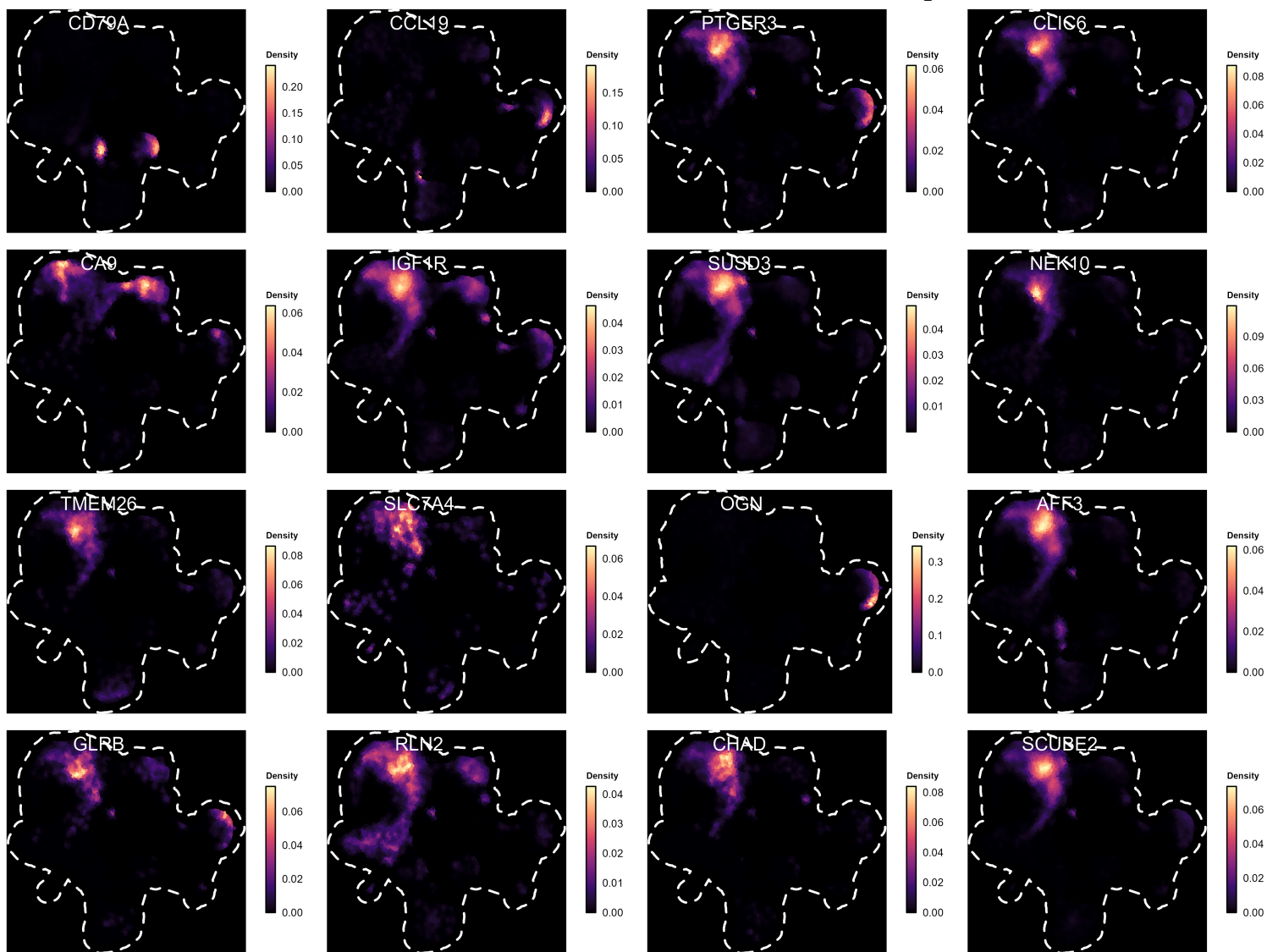

Supplement: Supplementary file 6 — Supplementary Material 6: Figure S6. A UMAP plot showing 36 cell clusters from 31 breast cancer patients. B UMAP plot showing cells derived from ER, HER2 or TNBC patients. Density plot of expression level of ECI genes in each cell [file 10020_2025_1237_MOESM6_ESM.pdf]

A

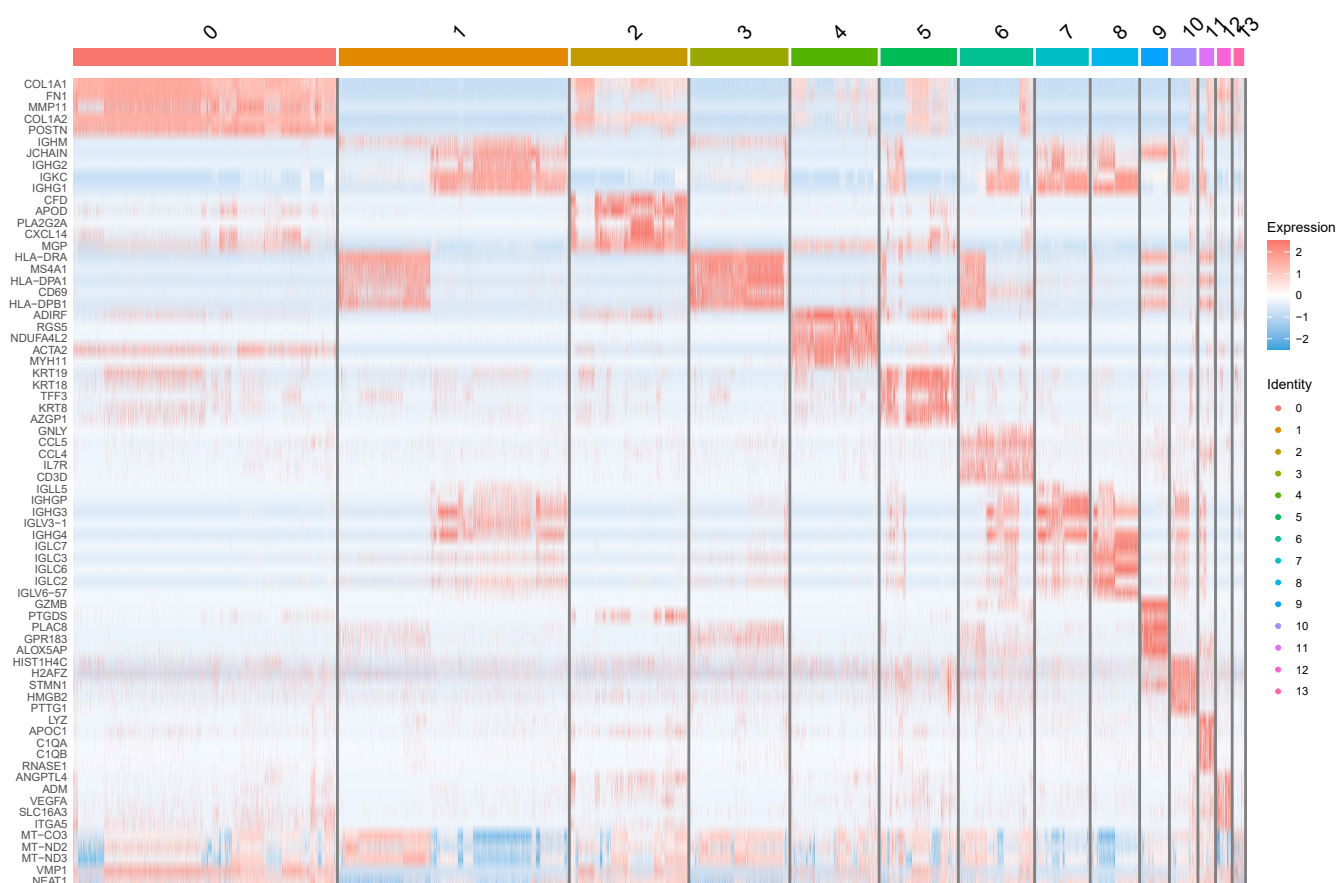

B

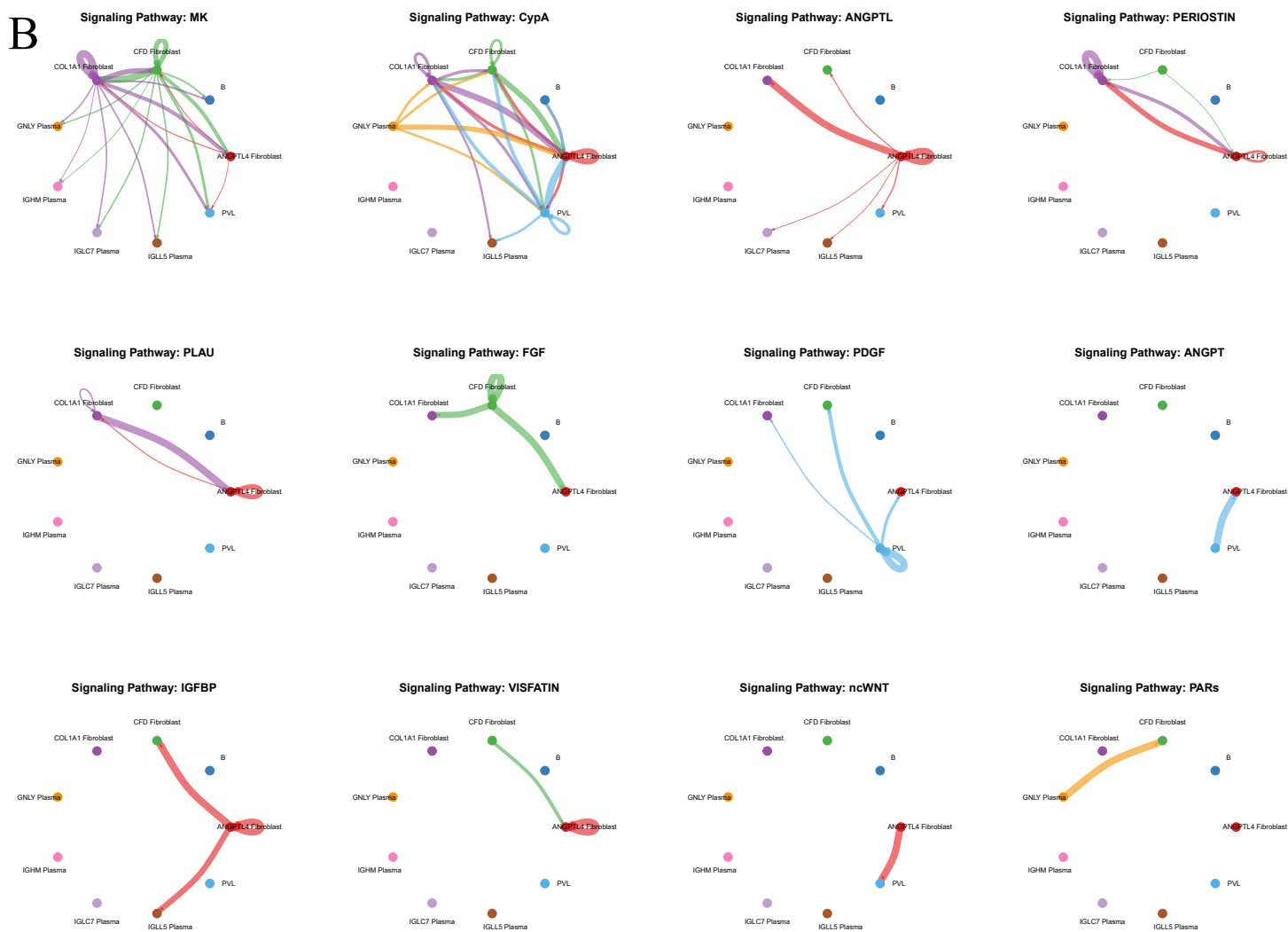

Supplement: Supplementary file 7 — Supplementary Material 7: Figure S7. A Heatmap plotting expression level of top 5 cell markers in each cell cluster of PVL, fibroblasts, and B-cell lineage. B Ligand-receptor interaction analysis among PVL, fibroblasts, and B-cell lineage [file 10020_2025_1237_MOESM7_ESM.pdf]
