# Supplementary material for: Transcriptome-wide analysis reveals potential roles of CFD and ANGPTL4 in fibroblasts regulating B cell lineage for extracellular matrix-driven clustering and novel avenues for immunotherapy in breast cancer
Source: Mol Med. 2025 May 8;31:179. doi: 10.1186/s10020-025-01237-y (PMC12063413; doi:10.1186/s10020-025-01237-y)
Supplement: Supplementary file 4 — Supplementary Material 4: Figure S4. A Heatmap plotting the results of CDI of various prognostic models. B RSF model selected from prognostic models. C Triplet graph demonstrating the distribution of patients, alive status, and expression level of ECI genes in the Training cohort. D Triplet graph demonstrating the distribution of patients, alive status, and expression level of ECI genes in the Testing cohort. E K-M curve plotting OS status of patients with NomogramHigh and NomogramLow in the Training cohort. F K-M curve plotting RFS status of patients with NomogramHigh and NomogramLow in the Training cohort. G K-M curve plotting OS status of patients with NomogramHigh and NomogramLow in the Testing cohort. H K-M curve plotting RFS status of patients with NomogramHigh and NomogramLow in the Testing cohort [file 10020_2025_1237_MOESM4_ESM.pdf]

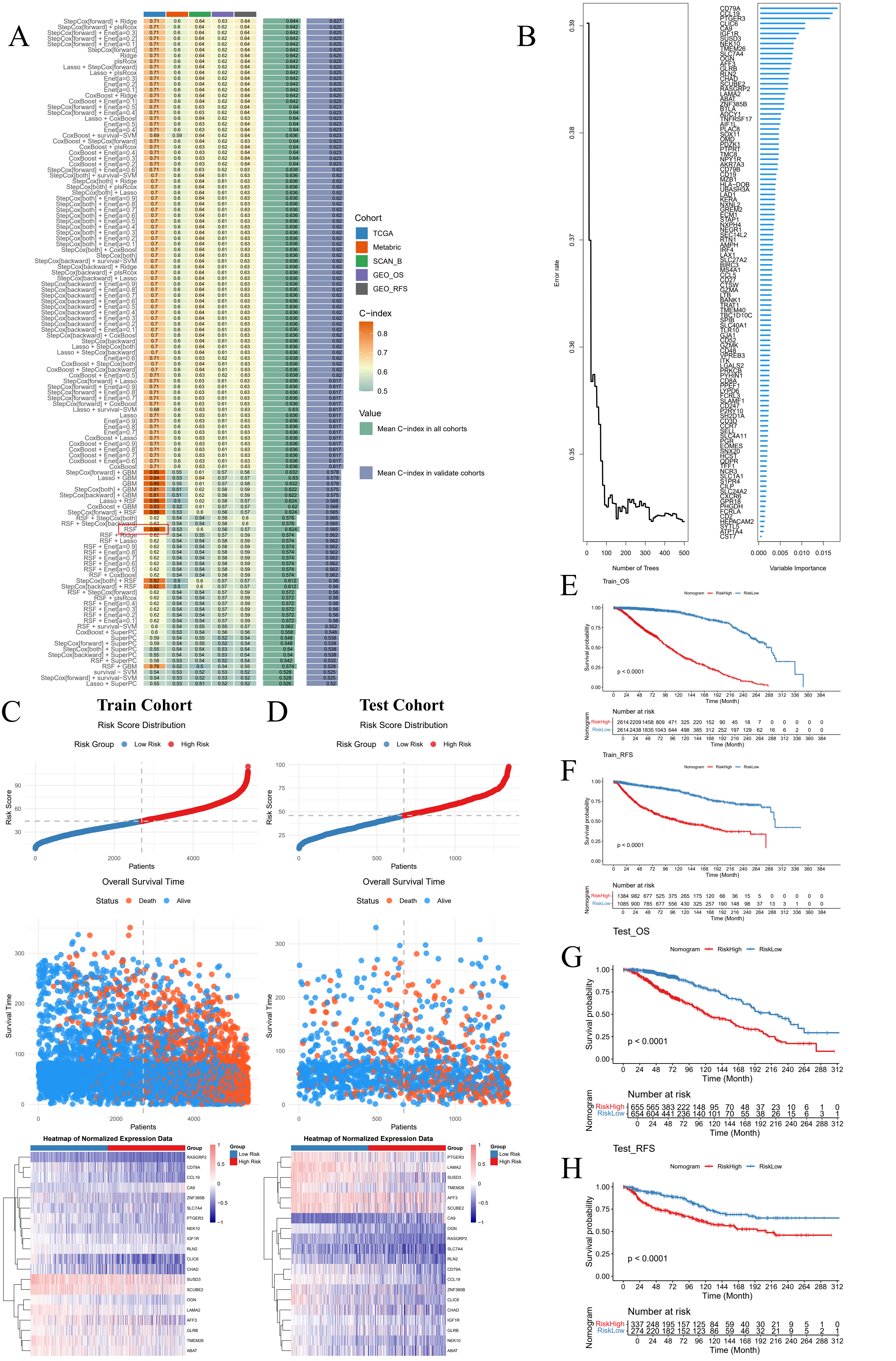

A
B
Cohort
TCGA
Metabric
SCAN_B
GEO_OS
GEO_RFS
C-index
0.8
0.7
0.6
0.5
Value
Mean C−index in all cohorts
Mean C−index in validate cohorts
Error rate
Number of Trees
Variable Importance
C
Train Cohort
Risk Score Distribution
Risk Group
Low Risk
High Risk
Risk Score
Patients
Overall Survival Time
Status
Death
Alive
Survival Time
Heatmap of Normalized Expression Data
Group
D
Test Cohort
E
Train_OS
F
Train_RFS
G
Test_OS
H
Test_RFS
Nomogram
RiskHigh
RiskLow
Survival probability
p < 0.0001
Time (Month)
Number at risk
